# Hypoxic and Fe‐Responses are Regulated by the ERFVII Factors and the PCO Branch of the N‐Degron Pathway According to Iron Availability

**DOI:** 10.1111/pce.70466

**Published:** 2026-03-08

**Authors:** Yuri Telara, Moez Maghrebi, Mikel Lavilla‐Puerta, Noemi La Monaca, Giulia Ambrogini, Alessio Sbrana, Sara Delucchi, Pierdomenico Perata, Gianpiero Vigani, Beatrice Giuntoli

**Affiliations:** ^1^ Institute of Plant Sciences Sant'Anna School of Advanced Studies Pisa IT; ^2^ Biology Department University of Pisa Pisa IT; ^3^ Department of Life Sciences and Systems Biology University of Turin Turin IT; ^4^ Department of Biology University of Oxford Oxford UK

**Keywords:** *Arabidopsis thaliana*, climate change, ERFVII, hypoxia, iron homeostasis, nutrient deficiencies, plant cysteine oxidases, protein stability, submergence

## Abstract

In plants, iron homeostasis and oxygen metabolism are strictly related, indeed several Fe‐requiring enzymes catalyze reactions that also involve O_2_ as a reagent, product, entry or end point of the pathway. Oxygen sensing itself relies on the Fe‐dependent enzymes Plant cysteine oxidases. However, the impact of iron deficiencies on the response to hypoxic stresses has not been investigated so far. PCOs channel the ERFVII ethylene‐responsive factors into a proteasomal N‐degron pathway that connects hypoxia‐inducible responses to the stabilization of the ERFVII transcription factors, which act as master regulators of plant hypoxic transcription. Here, we investigated the interplay between low oxygen and Fe‐deficiency stresses in *A. thaliana*. PCO activity *in vivo* was inferred from the expression of hypoxia marker genes and from the activity of a genetically encoded reporter of ERFVII protein stability. Our results highlight that Fe deprivation can elicit hypoxia‐like responses depending on its severity. Moreover, evidence from the pentuple *erfVII* mutant indicate that the ERFVIIs take part to the responses to chronic Fe‐deficiency and fine‐tune nutrient content to the shoot of submerged plants growing on moderately Fe‐deficient substrates. This work expands the known functions of the ERFVII factors and provides new information to understand plant responses to combined environmental stresses.

## Summary statement

In *A. thaliana*, Fe‐deficiency triggered hypoxia‐like responses via ERFVII stabilization. Under the combination of submergence and Fe‐deficiency, the ERFVIIs played a role in nutrient uptake and Fe‐responsive gene expression.

## 1 Introduction

Iron is an essential micronutrient for all living organisms, serving as a cofactor for numerous enzymes that regulate essential metabolic processes (Connorton et al. 2017; Vigani, Zocchi, et al. 2013). Plants tightly regulate iron content to sustain growth and development, and the homeostasis of this micronutrient ultimately impacts the quality and quantity of crop production (Kobayashi and Nishizawa 2012). Iron supply limitations in agriculture are generally faced with the adoption of integrated soil and crop management practices, to prevent the occurrence in crops of nutritional disorders associated with Fe‐deficiency (Mahender et al. 2019; Zuo and Zhang 2011). Indeed, despite the abundance of iron in the Earth's crust, its bioavailability is limited in nearly one‐third of the world's cultivated lands due to pH effects on soil redox state, which make iron less bioavailable to plants at neutral or alkaline pH (Manthey et al. 1994).

Fe uptake is enhanced through Strategy I, found in most flowering plants, or Strategy II mechanisms, restricted to grass species. Strategy I involves proteins like P‐type H + ‐ATPase 2 (AHA2), FRO2, and Iron Regulated Transporter 1 (IRT1) genes, which work together to increase iron solubility and uptake (Morrissey and Guerinot 2009). P‐type ATPase release protons into the rhizosphere, reducing the pH and increasing Fe solubility. Ferrous oxidoreductases (FROs) exhibit Fe Chelate Reductase activity (FCR), converting Fe^3+^ into Fe^2+^. This FCR activity is enhanced at acidic pH (Tsai and Schmidt 2017). Subsequently, ZIP transporters, such as IRT1 in *Arabidopsis thaliana*, facilitate the uptake of Fe^2+^ into plant roots. It has been demonstrated that AHA2, FRO2, and IRT1 form a protein complex at the root plasma membrane, which is suggested to create an optimal local environment with the right pH and Fe^2+^ concentration for efficient Fe uptake, preventing Fe^2+^ reoxidation in presence of oxygen in the soil (Martín‐Barranco et al. 2020). *Arabidopsis*, a Strategy I plant, also shares characteristics with Strategy II by secreting Fe‐mobilizing coumarins (FMC) from its roots into the rhizosphere. FMCs, derived from the phenylpropanoid metabolism, promote Fe uptake under conditions of low Fe availability (Robe et al. 2025).

The transcriptional regulation of Fe homeostasis in plants is a complex and critical process that governs proper acquisition, distribution, storage, and utilization of Fe while avoiding excessive accumulation that could lead to toxicity. In *A. thaliana*, Fe homeostasis is regulated by two interconnected transcriptional modules. The first module involves the Fer‐like Fe‐deficiency Induced Transcription factor (FIT/bHLH29) from clade IIIe (Colangelo and Guerinot 2004), which under Fe‐deficiency forms heterodimers with specific clade Ib TFs (bHLH38, bHLH39, bHLH100 and bHLH101) and activates the expression of *FRO2* and *IRT1* (Trofimov et al. 2019; Yuan et al. 2008; Wang et al. 2007) Conversely, clade IVa TFs (bHLH18, bHLH19, bHLH20, and bHLH25) repress FIT by promoting its degradation through the 26S proteasome pathway in a jasmonic acid‐dependent manner (Cui et al. 2018). The E3 ligases Brutus Like 1 (BTSL1) and Brutus Like 2 (BTSL2) are additional negative regulators of FIT, again targeting it to the proteasome (Rodríguez‐Celma et al. 2019). The second module involves the clade IVc bHLHs factors IAA‐Leucine Resistant 3 (ILR3), Iron‐deficiency Tolerant 1 (IDT1/bHLH34), bHLH104, and bHLH115 (Gao and Dubos 2021), which play additive roles in the response to Fe‐deficiency. Their functions are partially explained by the ability to form homo‐ or heterodimers that induce *FIT* expression. Additionally, Iron Man/Fe‐Uptake inducing peptides (IMA/FEP) participate to the Fe signaling cascade in *Arabidopsis*, with eight *IMA* genes regulating Fe homeostasis (Grillet et al. 2018). IMAs have been proposed to act as positive regulators of Fe uptake by regulating different components of Fe homeostatic control network. In particular, under Fe‐deficiency they can induce the expression of Ib clade bHLHs, *FRO2* and *IRT1* (Grillet et al. 2018; Hirayama et al. 2018). Also, IMA3 can inhibit BTS activity, buffering the degradation of ILR3 and bHLH115 proteins, and ultimately activating the Fe‐deficiency response (Li et al. 2021).

Such intricate regulatory mechanisms evolved to enable Fe homeostasis. The need to prevent both Fe‐deficiency and toxicity is linked to a central role of Fe chemistry in biological systems, established shortly after life appeared approximately 3.5 billion years ago, when Fe(II) was abundant in the anoxic Earth environment (Ilbert and Bonnefoy 2013). With the oxygenation of the Earth's atmosphere, Fe chemistry and biochemistry have become heavily influenced by the presence of oxygen (Ilbert and Bonnefoy 2013; Cammack et al. 1990). The functional relationship with oxygen can be incorporated in the classification of Fe‐requiring enzymes (FeREs), along with their function (Vigani and Murgia 2018). With few exceptions, such as aconitase and purple acid phosphatase, FeREs carry out redox reactions that in many cases also involve oxygen (Cammack et al. 1990).

Dioxygenases are a wide class of enzymes that catalyze reactions where both atoms of molecular oxygen are incorporated into one or more substrates (Kawai et al. 2014; Kundu 2012, 2015). Among the enzymes that use O_2_ as co‐substrate, those belonging to this class are almost exclusively FeREs. Plant dioxygenases employ different types of Fe cofactors to activate oxygen in a variety of reactions with organic substrates, using different catalytic mechanisms (Iacopino and Licausi 2020); (White and Flashman 2016). The largest subfamily is formed by 2‐OG/Fe‐dependent dioxygenases (2‐ODDs): their broad distribution in metabolism suggests that 2‐ODD requirement for Fe may underlie the widespread effects that Fe‐deficiencies have in plants (Farrow and Facchini 2014).

Among Fe‐dependent dioxygenases, Plant cysteine oxidases (PCOs) are 2‐OG independent enzymes that hold particular significance, as they function as genuine oxygen sensors (Gunawardana et al. 2022; Weits et al. 2014; White et al. 2018). PCOs affect the stability of group VII ethylene response factors (ERFVIIs), which are responsible for the fast activation of a core set of hypoxia‐inducible genes conserved in plants (Abbas et al. 2015; Gasch et al. 2016). PCOs employ a mononuclear Fe(II) center to catalyze Cys‐sulfinic acid formation at the N‐terminal end of the ERFVII transcription factors, using O_2_ as co‐substrate. Cysteine oxidation turns the ERFVIIs into targets for arginylation via ATE1/2, ubiquitination via the E3‐ligase enzyme PRT6 and eventual degradation via the 26S proteasome (Gibbs et al. 2011; Licausi et al. 2011; White et al. 2017). Such proteolytic mechanism connecting protein half‐life to O_2_ concentration is also known as cysteine or PCO branch of the N‐degron pathway. PCO inactivation under hypoxia blocks the Cys N‐degron pathway, leading to ERFVII accumulation and the induction of the hypoxicgenes. Recently, a similar mechanism has been discovered in humans, where hypoxic responses are promoted by the homologous thiol dioxygenase ADO (Iacopino and Licausi 2020; Masson et al. 2019).

Iron homeostasis and O_2_ metabolism are known to be linked in plants. Fe‐deficiency‐induced responses are often associated with increased oxygen consumption rates in roots (López‐Millán et al. 2000; Vigani et al. 2009), whereas in leaves lower oxygen consumption and evolution have been described (Vigani et al. 2017). Despite these pieces of evidence, there is limited information regarding the specific impact of Fe‐deficiency on the dynamics of oxygen responses at the cellular level. The main goal of this study was to provide new insights in the interplay between Fe‐deficiency and hypoxic regulation in plants, stemming from the observation that O_2_ sensing relies on the Fe‐requiring PCO enzymes.

## 2 Methods

### 2.1 Plant Material and Growth Conditions

The Columbia‐0 ecotype (Col‐0) of *A. thaliana* was used as the wild type background in all experiments. The pentuple mutant *erfVII* (Abbas et al. 2015) and the transgenic lines *28RAPFluc (35S:RAP2.12*_*1‐28*_ *‐FLUC)* (Weits et al. 2014) and *Δ13RAP2.12* (*35S:RAP2.12*_*4‐358*_) (Licausi et al. 2011) have been described previously. The mutants *pco1/2* (Weits et al. 2014) *pco4/5* (Weits et al. 2023) *fit1‐2* (N62602; Colangelo and Guerinot 2004) and *uri1* (N684684; Kim et al. 2019) were described previously. For axenic experiments, seeds of *A. thaliana* were surface sterilized with 70% (v/v) ethanol and 10% sodium hypochlorite and germinated on half‐strength MS medium (Duchefa) supplemented with 10 g L^−1^ sucrose and 9 g L^−1^ agar (Duchefa). Plants were grown with 23°C day/19°C night temperature and a 12 h light period with a light intensity of 120 μmol photons m^−2^ s^−1^. Soil experiments were carried out according to (Murgia et al. 2015).

### 2.2 Cloning of Constructs

Plasmids pAG415‐PCO4 and pAG415‐GUS, used for yeast transformation, have been described before (Puerta et al. 2019). The chimeric DLOR‐bHLH039 sequence, inspired to DLOR‐RAP2.12 (Puerta et al. 2019) was designed by replacement of the original RAP2.12_2‐28_ sequence with bHLH039_2‐50_, and purchased as a synthetic Gateway^TM^‐compatible (CACC‐starting) DNA string from GeneArt (Thermo Fisher Scientific). The insert was cloned into pENTR‐D/TOPO (Thermo Fisher Scientific) and subsequently recombined in the pAG413GPD‐ccdB destination vector (Addgene plasmid #14142; Alberti et al. 2007) using the LR Clonase II Enzyme Mix (Thermo Fisher Scientific), according to the manufacturer's recommendations.

The full length coding sequence of *bHLH039* was amplified from *Arabidopsis* cDNA, obtained as described in the paragraph “Gene expression analyses”. To amplify Gateway^TM^‐compatible inserts with, respectively, Cys2 or Ala2 sequence, primers bHLH039(C)gw_FW (5'‐CACCATGTGTGCATTAGTACCT‐3’) or bHLH039(A)gw_FW (5'‐CACCATGGCTGCATTAGTACCT‐3’) were combined with primer bHLH039_RV (5’‐TATATATGAGTTTCCACATTCC‐3’). Amplifications were carried out with the high‐fidelity Phusion DNA polymerase (Thermo Fisher Scientific), following the manufacturer's recommendations. Fragments were cloned with the Gateway^TM^ system in the non‐binary destination vector p2GWL7 (Weits et al. 2014) obtained by ligation into the p2GW7 backbone of an ApaI/SpeI fragment excised from vector pBGWL7 both from Karimi et al. (2002).

### 2.3 Iron‐Deficiency Treatments

Nutrient composition of the modified MS media used for axenic experiments was described in detail by (Gruber et al. 2013). Fe‐depletion treatments were performed by iron exclusion from the liquid media. Additionally, the agar was soaked in a solution containing 2 mM CaSO_4_ and 10 mM EDTA (pH 8) for 30 min and washed three times with pure H_2_O, each wash lasting 8 h. Fe‐chelation treatments were performed by supplementation of full nutrient media with 300 µM 2,2’‐bipyridyl (Sigma‐Aldrich) dissolved in DMSO (final DMSO concentration, 0.1% v/v); control samples here were treated with an equal dose of DMSO.

For short‐term Fe‐deficiency treatments, 7‐day‐old *28RAPFluc* seedlings were transferred from vertical plates to 6‐well plates containing freshly prepared liquid media containing the specified iron concentration. Before the transfer, seedlings were kept in iron wash solution for 30 min, to remove iron from the apoplast, and rinsed with sterile distilled water.

In soil experiments, alkaline conditions were imposed according to Murgia et al. (2015). Control soil had pH 5.5, whereas alkaline soil (pH 7.8) was prepared by CaO supplementation (8 g CaO kg^−1^ substrate). The soil was moistened and thoroughly hand‐mixed, its pH was measured after few hours and adjusted with further supplements of CaO, when needed. Soil was then mixed again and its pH measured several times in the next 2 days and each time adjusted to the target pH with CaO, before use. The pH was measured again at the end of the experiments, never exceeding 0.2 pH units drop.

### 2.4 Low Oxygen Treatments

Hypoxic treatments were carried out in a Gloveless Anaerobic chamber (COY). Plants were treated in the dark with 1% O_2_ (v/v) atmosphere, or normoxic atmosphere, for the specified duration, at 23°C constant temperature. Dark submergence on 25 day‐old soil grown plants was protracted for 12 h, covering plants with 10 cm tap water column, pre‐equilibrated at room temperature. Yeast cultures were treated for 6 h with 1% O_2_ (v/v) or normoxic atmosphere.

### 2.5 Analysis of Main Root Length

Primary root length of individual *Arabidopsis* seedlings was measured with ImageJ (Schneider et al. 2012). Plate images were taken at the end of the treatment period, using an Epson Expression 10000XL scanner (Seiko Epson) in color at 600 dpi resolution. Tests of statistical significance were undertaken with GraphPad 8.0 (Prism) using a two‐way ANOVA with pairwise comparisons through the Tukey post‐hoc test.

### 2.6 Yeast Culture and Treatment

The heterologous assays were carried out in the *S. cerevisiae* strain BY4742 (*Matα; his3‐Δ1; leu2‐Δ0; lys2‐Δ0; ura3‐Δ0*) (Scientific Research and Development GmbH). Untransformed cells were grown on YPDA (20 g L^−1^ peptone, 10 g L^−1^ yeast extract, 20 g L^−1^ of glucose, 20 g L^−1^ agar, Duchefa). To generate the reporter strains, the pAG413‐DLOR‐bHLH039 plasmid was transformed along with pAG415‐PCO4 or pAG415‐GUS according to the LiAc/SS carrier DNA/PEG method, as previously described (Lavilla‐Puerta et al. 2023). Transformed cells were grown at 30°C in SD medium (6.7 g L^−1^ Yeast Nitrogen Base DIFCO, 1.37 g L^−1^ Yeast Dropout Medium, 20 g L^−1^ glucose), plus adequate supplements (0.16 M uracil, 0.8 M histidine–HCl, 0.8 M leucine and 0.32 M tryptophan, when complete) and 20 g L^−1^ agar.

For the assay, five independent colonies were inoculated in 200 μl liquid SD medium with selection, in flat bottom 96‐well polystyrene plates, in static regime. Overnight cultures were diluted to the early exponential phase with fresh media, allowed to resume growth and then adjusted to OD_600_ = 0.02 prior to treatment. The optical density of the cultures was measured directly in the microplate with a Multiskan Go 1510 Sky plate reader (Thermo Fisher Scientific). The cultures were then grown for 6 h under normoxic or hypoxic atmosphere, as illustrated above, with manual pipette mixing once in 2 h. At the end of the treatment, cells were recovered by centrifugation and subjected to luciferase assay.

### 2.7 Protoplast Transformation

*Arabidopsis* protoplasts were isolated from mesophyll cells and transfected as described in(Weits et al. 2014). In every independent transformation, 100 μl protoplast suspension, containing approximately 2 × 10^5^ cells, were co‐transformed with 5 μg reporter plasmid (p2GW7‐bHLH039 or p2GW7‐RAP2.12_1‐28_Fluc) and 2 μg p2GW7‐Rluc normalizer plasmid. After 16 h incubation in the dark, protoplasts were recovered by gentle centrifugation and flash‐frozen for the subsequent luciferase assays.

### 2.8 Luciferase Assays

Luciferase activity in *28RAPFluc* seedlings and in yeast microcultures was quantified using the Dual‐Luciferase Reporter (DLR) Assay System (Promega) and a Lumat LB 9507 Tube Luminometer (Berthold), as described in (Lavilla‐Puerta et al. 2023). For plant samples, values were normalized to the total protein amount, as determined through the Bradford protein assay (Bio‐Rad), while for yeast samples values were expressed as Fluc/Rluc ratio.

### 2.9 Immunoblotting

Total proteins were extracted from 7‐day‐old *Arabidopsis* seedlings with 400 μL extraction buffer (50 mM Tris‐HCl pH 7, 1 mM EDTA pH 8, 100 mM NaCl, 2% SDS, and 0.05% Tween‐20 with a Protease Inhibitor Cocktail from Sigma). Extracts were centrifuged at 8000 g for 10 min at 4°C, and the protein concentration in the supernatant was measured using the Pierce BCA Protein Assay Kit (Thermo Fisher Scientific). 50 μg protein extract were denatured at 95°C for 5 min in 0.8 M DTT and XT Sample Buffer (Bio‐Rad) and separated by SDS‐PAGE on 10% polyacrylamide gels (NuPage Bis‐Tris Gels, Thermo Fisher Scientific). Proteins were blotted onto a PVDF membrane (Bio‐Rad) using the TransBlot® Turbo transfer system (Bio‐Rad). For PCO1‐GFP immunodetection, a monoclonal anti‐GFP antibody (11814460001, Roche, 1:3000 dilution) was used and revealed with an anti‐mouse IgG HRP (Agrisera AS09 627; 1:20000 dilution). An anti‐HA peroxidase conjugated antibody (3F10, Roche, 1:1000 dilution) was used for the immunodetection of RAP2.3^3xHA^. Actin‐11 was detected with a polyclonal primary antibody (AS13 2640, Agrisera, 1:5000 dilution) coupled with a goat anti‐rabbit IgG HRP secondary antibody (Agrisera AS09 602; 1:15000 dilution). All antibodies were diluted in 4% skim milk solution in PBST. Signal detection was performed with Clarity Max Western ECL Substrate (Bio‐Rad), using a ChemiDocTM MP Imaging System (Bio‐Rad).

### 2.10 Gene Expression Analyses

Total RNA was extracted as previously described (Kosmacz et al. 2015). RNA integrity was checked by gel electrophoresis on 1% (w/v) agarose, followed by spectrophotometric quantification. Reverse‐transcription was performed with the Maxima First Strand complementary DNA (cDNA) Synthesis Kit (Thermo Fisher Scientific), following the manufacturer's recommendations. RT‐qPCR was performed with an ABI Prism 7300 sequence detection system (Applied Biosystems), using 12.5 ng cDNA template PowerUp SYBR® Green Master Mix (Thermo Fisher Scientific). *UBQ10* (*AT4G05320*) housekeeping gene expression was deployed for the calculation of relative gene expression following the ΔΔCt method (Livak and Schmittgen 2001). Primer sequences are specified in Table S1.

### 2.11 ICP‐MS Measurements

Tissues (rosette leaves or entire seedlings) of plants were washed with Milli Q water and dried in a ventilated oven at 70°C for 4 days. The dry weights were measured, and tissues were then digested with 500 µL 65% HNO_3_ for 4 h at 120°C. Once mineralized, 250 µL of 65% HNO_3_ was added, and vortexed samples were transferred into polypropylene test tubes with a 1:40 dilution with Milli‐Q water. Finally, the mineral contents of the samples were measured by inductively coupled plasma‐mass spectrometry ICP‐MS (BRUKER Aurora‐ M90 ICP‐MS), as previously described (Vigani et al. 2021). The analyses were carried out from three independent biological replicates.

## 3 Results

### 3.1 Plant Cysteine Oxidase Activity Changes in Iron‐Starved Plants

Since PCOs are Fe‐dependent enzymes, we speculated that they may function as a convergence point between hypoxic signaling and Fe‐deficiency signaling. To investigate the effect of different Fe provisions on PCO activity, we deployed the *A. thaliana* reporter line *35S:RAP2.12*_*1‐28*_ *‐Fluc*, expressing an ERFVII‐based translational fusion (herafter indicated as 28RAPFluc) that informs about the activity of the Cys N‐degron pathway (Lavilla‐Puerta et al. 2023; Weits et al. 2014). In this line, firefly luciferase abundance is subjected to Cys2 regulation on the construct, but unaffected by mechanisms intervening on different domains of RAP2.12 beyond its N‐terminus, such as signaling of the energy status (Weits et al. 2019) or proteolysis through SINAT1/2 E3 ligases (Papdi et al. 2015).

**Figure 1:**
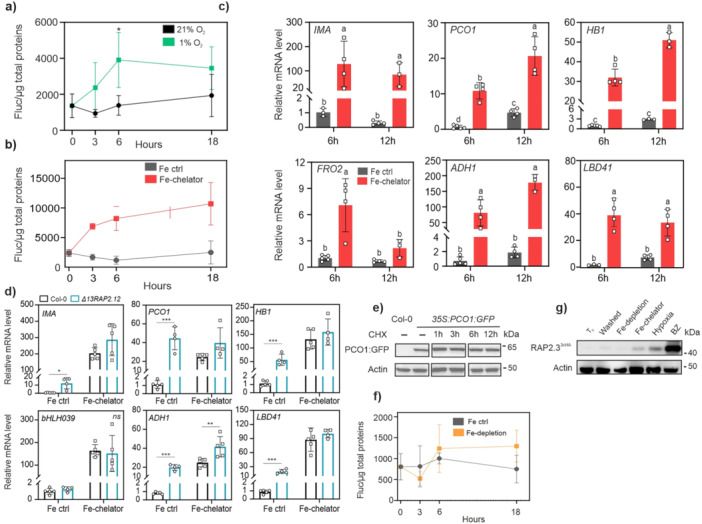
Modulation of hypoxic responses under different Fe‐removal treatments. (a) Response of liquid grown 5 day‐old *35S:RAP2.12*_*1‐28*_ *‐Fluc* seedlings to hypoxia. Luciferase activity was measured at different time points of dark hypoxia (1% O_2_ v/v) or dark normoxia and normalized with soluble proteins. Data in (a), (b) and (e) are mean ± SD (*n* = 5). (b) Effect of Fe‐chelation on the 28RAPFluc reporter. Seedlings were transferred from vertical plates to 300 µM 2,2‐dipyridyl (DIP), or to a control medium with 0.1% DMSO (v/v, “Fe‐control”), and luciferase activity was measured after 3, 6 and 18 h treatment in the dark. (c) Hypoxia marker gene expression under Fe‐chelation conditions. Five day‐old wild type seedlings, grown in liquid media, were treated with 300 µM DIP for 6 or 12 h in darkness, or with an equivalent amount of DMSO (“Fe‐control”). The Fe deficiency‐inducible gene *IRONMAN* was included to monitor the effects of Fe‐chelation. Data are mean ± SD (*n* = 5). Letters indicate statistically significant difference (*p* < 0.05, two‐way ANOVA, Tukey–Kramer post‐hoc test). (d) Marker gene expression in *35S:Δ13RAP2.12* seedlings treated as in (c), after 6 h DIP treatment. Asterisks mark statistically significant differences (*p* < 0.05, Tukey *t*‐test, *n* = 5). (e) Immunoblotting of *35S:GFP:PCO1* seedlings over a time course of cycloheximide treatment (CHX, 200 µM). (f) Effect of Fe‐deficiency on 7 day‐old *35S:RAP2.12*_*1‐28*_ *‐Fluc* plants. Seedlings grown on full media in vertical plates were shifted to fresh Fe‐deficient (“Fe‐depletion”) or full liquid medium (“Fe control”) after apoplast wash, and treated under neutral photoperiod. t_0_ corresponded to 4 p.m. Pairwise comparisons at every time point showed no significant differences (*p* > 0.05). (g) Immunoblotting of RAP2.3 amount in *35S:RAP2.3* ^*3xHA*^ seedlings, 7 day‐old, grown in vertical plates and treated for 6 h in the dark. t_0_, before apoplast wash; washed, immediately after wash; Fe‐depletion, Fe deficient medium; Fe‐chelator, 300 µM DIP; hypoxia, 1% O_2_ (v/v) atmosphere; BZ, 300 µM bortezomib. ACT, actin‐11 housekeeping protein. Supporting blots are provided in Figure S1. [Color figure can be viewed at wileyonlinelibrary.com]

Preliminarily, we tested the responsivity of the reporter line to PCO inactivation by imposing hypoxia to 7‐day‐old seedlings, under Fe‐replete conditions. In aerated samples, the signal remained unaltered over 18 h darkness, while it increased gradually in response to 3 and 6 h oxygen deprivation (dark hypoxia), reaching a maximum of 2.8‐fold induction, with no further increase at 18 h (Figure 1a). We compared the output range under hypoxia, assumed as a strong inhibitory treatment of PCO activity, with 28RAPFluc response when Fe levels were manipulated under aerobic conditions. Complete removal of Fe from the apoplast and media, after supplementation of the Fe‐chelating compound bipyridyl, caused up to 4.5‐fold stabilization of the reporter (Figure 1b). The response had a similar range to the one following acute hypoxia, but with faster and prolonged dynamics. ERFVII stabilization was accompanied by strong induction of downstream transcriptional responses, according to the hypoxic markers *Alcohol dehydrogenase 1* (*ADH1*), *Lateral organ Binding Domain 41* (*LBD41*) and *PCO1* (Figure 1c). Transgenic *Arabidopsis* plants overexpressing a Δ13RAP2.12 truncation (ΔRAP2.12, Licausi et al. 2011) show constitutive activation of hypoxic transcription. Here, no further induction of the hypoxic markers could be stimulated by bipyridyl, indicating that the N‐terminal domain is essential and sufficient for the response (Figure 1d). These observations suggest that plant PCOs can be readily and effectively inactivated by harsh Fe‐chelation treatments that make free Fe^2+^ unavailable. In parallel, we monitored the half‐life of a GFP‐tagged PCO1 version, stably expressed by *35S:PCO1:GFP* plants (Weits et al. 2014) treated with the protein synthesis inhibitor cycloheximide (CHX). The immunoblotting showed that the fusion protein was characterized by long half‐life (Figure 1e and Figure S1a). The slow turnover rate of PCO1 suggests that the iron cofactor was subtracted to previously formed PCOs, rather than made unavailable to newly synthesized enzymes.

To simulate conditions closer to physiological Fe‐deficiencies, we imposed a milder treatment consisting in Fe removal from the apoplast by washing, followed by the transfer of seedlings on Fe‐free media. In this case, Fe‐depletion under photoperiodic conditions did not promote any stabilization of the 28RAPFluc reporter over 18 h treatment (Figure 1f). No reporter induction was observed when Fe depletion was combined with continuous darkness (Figure S2a), ruling out that the exposure to light might interfere with the stabilization of the reporter in the previous experiment. We used the hemagglutinin‐tagged line *35S:RAP2.3* ^*3xHA*^ (Gibbs et al. 2014) to visualize ERFVII abundance under iron‐deficiency by immunoblot. We could confirm that short‐term Fe‐depletion had no consequences on protein degradation, whereas Fe‐chelation was comparable with 1% O_2_ (v/v) treatment in restraining RAP2.3 turnover (Figure 1g and Figure S1b). Proteasome inhibition after bortezomib (BZ) supplementation predictably had the strongest effect, due to fast and complete arrest of RAP2.3 degradation.

Longer Fe‐depletion treatment, spanning over 48 h, was not able to promote 28RAPFluc stabilization either (Figure S2b). In the same conditions, the hypoxia marker genes tested *PCO1, ADH1, PDC1* (*Pyruvate decarboxylase 1*) and *SAD6* (*Stearoyl‐acyl carrier protein Δ9‐desaturase 6*) showed sporadic and minimal responses to Fe‐depletion, which were mostly recorded after the last time point (Figure S2c). Such late induction appears to be unrelated to the early signaling events expected to follow PCO inactivation. Altogether, the previous observations indicated that transient Fe deprivation conditions do not generally associate with RAP2.12 stabilization. In contrast, Fe‐chelation achieved by bipyridyl treatments caused fast and significant stabilization of the ERFVII factors.

### 3.2 Cys N‐Degron Pathway Impact on the Proteostasis of MC‐Containing Ib bHLH Transcription Factors

**Figure 2:**
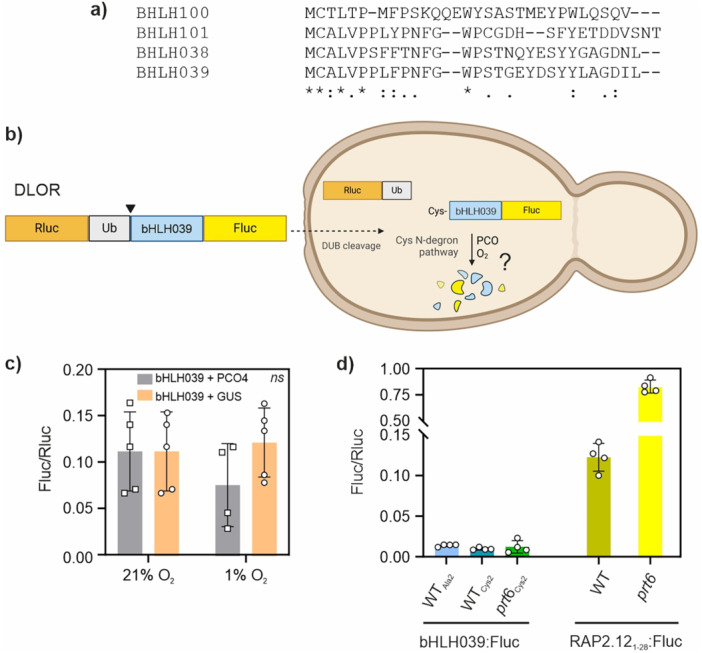
Subgroup Ib bHLHs turnover in relationship with the Cys N‐degron pathway. (a) N‐terminal sequence alignment of the Cys2‐containing subgroup Ib bHLH proteins of *Arabidopsis*. (b) Post‐translational processing of the ratiometric reporter DLOR‐bHLH039 in yeast. Once expressed in yeast cells, the chimeric protein is cleaved by native deubiquitinating enzymes (DUBs), which release a Cys‐exposing bHLH039 N‐terminal fragment fused to firefly luciferase (Fluc) and the renilla luciferase protein (Rluc, used for normalization). Expression of heterologous PCO enzymes (here, PCO4 from *A. thaliana*) makes Cys‐exposing substrates O_2_‐labile through the native Arg N‐degron pathway of yeast. Non‐substrate proteins, instead, will remain stable. (c) Output of the DLOR‐bHLH039 reporter, after 6 h treatment with hypoxia (1% O_2_ v/v) or normoxia (21% O_2_ v/v), in yeast cultures expressing AtPCO4 or a negative control construct (GUS, β‐glucoronidase). Values are mean ± SD (*n* = 4–5). Two‐way ANOVA and Tukey–Kramer post‐hoc test showed no significant differences (*p* > 0.05). (d) Fluc activity of the bHLH039‐Fluc reporter and the 28RAPFluc control construct (RAP2.12_1‐28_‐Fluc) expressed in *Arabidopsis* mesophyll protoplasts, normalized on total protein content in the extracts. Data are mean ± SD (*n* = 4). WT, Col‐0. Cys2, wild type *bHLH039* coding sequence; Ala2, Cys2Ala substituted version. [Color figure can be viewed at wileyonlinelibrary.com]

A potential hub between O_2_ and iron signaling is represented by the Fe‐inducible subgroup of clade Ib bHLH transcription factors, composed of bHLH038, 039, 100 and 101 in *A. thaliana* (Wang et al. 2007), due to their conserved Cys2 residue (Figure 2a). Such observations led us to speculate of a functional role for Cys2 in making Fe‐inducible clade Ib bHLH transcription factors susceptible to the Cys N‐degron pathway.

We chose bHLH039 as representative candidate from the set of four closely related proteins (Gao and Dubos 2024). To evaluate its susceptibility to PCO‐dependent degradation, we first resorted to an established reporter assay strategy deploying baker's yeast (*Saccharomyces cerevisiae*) as heterologous testbed. We expressed a ratiometric luciferase‐based reporter in yeast, DLOR‐bHLH039, inspired to the one described in Puerta et al. (2019). DLOR‐bHLH039 is a ubiquitin‐fusion construct that, upon cleavage by endogenous deubiquitinating enzymes, releases a Cys‐exposing fragment corresponding to the bHLH039_2‐50_‐Fluc sequence (Figure 2b). In this way, firefly luciferase (Fluc) activity provides an *in vivo* proxy of bHLH039 susceptibility to the N‐degron pathway. In yeast cultures, DLOR‐bHLH039 stability was unchanged regardless of AtPCO4 expression and atmospheric oxygen availability (Figure 2c). The substrate appeared to be very unstable in both tested conditions, hinting at PCO‐independent degradation phenomena impinging on the protein fragment.

The absence of regulation may be due to artefacts associated with yeast‐specific regulation, or to the lack of essential sequence features downstream of the Ala50 residue of bHLH039 (such as native Lys residues for ubiquitin conjugation, or possible domains participating to the interaction with PCOs). To exclude these events, we proceeded to evaluate the stability of the full‐length bHLH039 protein in isolated mesophyll protoplasts of *Arabidopsis*. While the ERFVII reporter construct 28RAPFluc was markedly stabilized in the *prt6* mutant (impaired in the last step of the Cys N‐degron pathway), the full‐length bHLH039‐Fluc fusion protein was unaffected by either PRT6 inactivation or Cys2Ala substitution that would prevent PCO action (Figure 2d). Altogether, we could therefore rule out bHLH039 as a substrate of the Cys N‐degron pathway. Moreover, the fusion protein emitted very low luminescence, suggesting that, in leaf cells, bHLH039 may be subjected to post‐translational regulation to maintain protein levels low in Fe‐replete conditions.

### 3.3 Role of the ERFVII Factors in the Responses to Prolonged Fe‐Deficiency

We showed before that the PCO branch of the N‐degron pathway can be hampered in plants exposed to Fe‐deficiency, leading to a reduction in ERFVII protein turnover (Figure 1). To delve more into the interplay between the Cys N‐degron pathway and iron physiology, we examined the expression of ERFVII‐regulated genes under chronic Fe starvation. Nutritional stresses were applied both to young *Arabidopsis* seedlings, germinating them on Fe‐deficient axenic media, and rosette‐stage plants, cultivated on alkaline soil (pH 7.8). Fe responses were monitored with the use of marker genes that are modulated by Fe‐deficiency regardless of plant tissues (*bHLH038, bHLH039, IMA*), along with *FRO* genes (Rodríguez‐Celma et al. 2019).

Wild type seedlings grown on plates displayed variable iron‐deficiency symptoms across experiments. This outcome was not unexpected, since previous reports indicate that, for most elements, consistent levels of nutrient deficiency are difficult to reproduce when solid synthetic media are used (Gruber et al. 2013). The variability was likely due to persistence of traces of iron contaminant in the agar powder, after the agar washing procedure adopted. Although equal iron depletion over replicate experiments could not be achieved, this situation nonetheless gave us the chance to monitor plant responses under slightly different starvation conditions, at near‐zero Fe levels.

**Figure 3:**
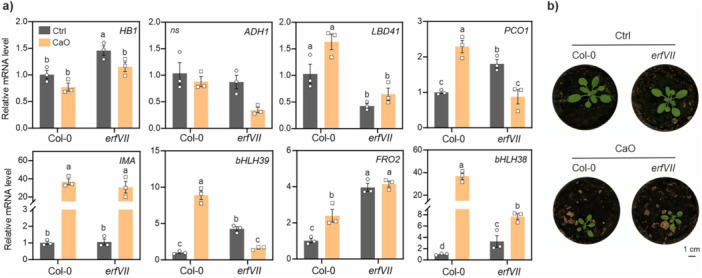
Response to chronic Fe‐deficiency in rosette stage plants. (a) Expression of hypoxic and Fe‐starvation markers in rosette tissues of Col‐0 wild type or *erfVII* mutant plants cultivated for three weeks on regular (Ctrl, pH=5.5) or alkaline substrate ( + CaO, pH=7.8). (b) Representative pictures of plants used for the analysis. [Color figure can be viewed at wileyonlinelibrary.com]

In seedlings, permissive Fe‐deficiency was indicated by limited chlorosis, mild growth inhibition (Figure S3a,b), and moderate upregulation of Fe‐deficiency markers in the wild type (Figure S3c). Interestingly, no hypoxic gene induction took place in these conditions (Figure S3c). The hypoxic response was also absent in rosette tissues of wild type plants cultivated on moderate alkaline soil for 3 weeks (Figure 3a). Supplementation of calcium oxide to the substrate increased soil pH from 5.5 to 7.8, causing a decrease in plant growth (Figure 3b). The small induction of the Fe‐deficiency markers, along with weak chlorosis symptoms, suggested that these plants experienced moderate nutritional stress, similar to axenic seedlings above. This combined evidence suggests that plants affected by moderate Fe‐deficiency for prolonged time did not generally invoke any long‐lasting hypoxic signaling. We cannot rule out that transient hypoxic responses may have occurred at earlier growth stages, in starved seedlings or rosettes. However, our previous experiments with short‐term treatments, failing to reveal any activation of low oxygen markers (Figure 1f and Figure S2), rather support the conclusion that hypoxic signaling is not elicited by mild Fe‐starvation. Defective genotypes in Fe acquisition, such as the *fit1‐2* and *uri1* mutants, grown on Fe‐replete media also showed no up‐regulation of the anaerobic markers (Figure S4), compatible with the conclusion that chronical suboptimal levels of tissue Fe is not enough to inhibit the Cys N‐degron pathway.

**Figure 4:**
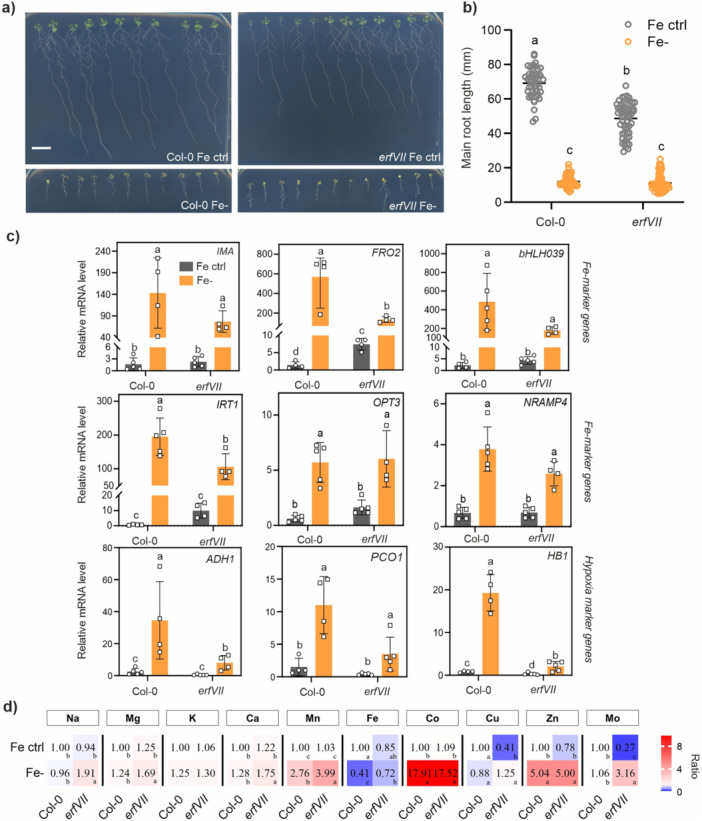
Response to chronic Fe‐deficiency in *Arabidopsis* seedlings. (a) Representative pictures of wild type and *erfVII* seedlings grown for 10 days on control or Fe‐plates. Five replicate plates were observed for each experimental thesis. (b) Primary root length of plants in (a) (*n* = 57). (c) Expression of Fe‐deficiency, Fe transport and hypoxia markers in full seedlings from the same experiment. Expression values (mean ± SD, *n* = 5) are presented as normalized to a wild type control sample. Distinct letters indicate statistically significant differences after two‐way ANOVA and Tukey–Kramer post‐hoc test (*p* < 0.05). (d) Heatmap of relative ion concentrations in seedlings, normalized on the average ion concentration in control Col‐0 plants. Different letters indicate significant differences between conditions and lines (two‐way ANOVA, Tukey's test, *p* < 0.05, *n* = 3). Raw data are provided in Table S2. [Color figure can be viewed at wileyonlinelibrary.com]

Seedlings experiencing severe Fe‐deficiency displayed, instead, extremely stunted growth (Figure 4a,b), accompanied by strong upregulation of Fe‐starvation markers still observable 10 days after germination (Figure 4c). Different from before, these harsh conditions strongly stimulated the induction of hypoxic markers (Figure 4c). The observed hypoxia‐like response indicates that extreme chronic deprivation of Fe can lead to a prolonged impairment of PCO activity in plants.

A pentuple *erfVII* mutant was included in the same experiments, to investigate how the modulation of the PCO pathway invoked by the stress may connect with physiological responses to Fe‐deficiency. In adult Fe‐starved plants (Figure 3a) and, marginally, in seedlings (Figure 4c), the upregulation of *bHLH039* was lower in the *erfVII* mutant than the wild type. The consistent trend of transcriptional regulation of Fe‐ markers shown by plants at two distinct growth stages and from different substrates suggests that the ERFVIIs may take part to the acclimation to chronic Fe‐deficiency throughout plant life, by upregulating a subset of starvation‐responsive genes.

We evaluated whether the observed transcriptional regulation impacted *erfVII* ability to cope with strong iron starvation at the seedling stage. After ten days on control media, the mutant produced significantly shorter primary roots than the wild type, in line with its previous phenotypic characterization (Shukla et al. 2019). Extreme Fe‐deficiency, instead, prevented acclimation, causing the inhibition of root elongation in both genotypes (Figure 4a, b).

To gain insights in the physiological status of moderately starved seedlings, we determined the ionome profile of plants tissues by ICP‐MS. We found no substantial differences in macronutrient content across genotypes and conditions, except for slightly higher levels of Ca and Mg in Fe‐ *erfVII* seedlings (Figure 4d and Table S2). On the contrary, Fe deficiency markedly favored micronutrient and metal ion (Co) intake, resulting in more elevated levels of three out of five micronutrients measured (Mn, Cu and Zn). The ion profiles of Fe‐starved plants were similar between genotypes, but significant interaction between variables was detected in the case of some micronutrients (Mn, Cu and Mo, whose homeostasis was completely altered in the *erfVII*) (Figure 4d and Table S2), suggesting a role for the ERFVIIs in the fine‐tuning of micronutrient content during chronic Fe‐deficiencies. The most remarkable difference between genotypes was, however, represented by Fe itself, more scarce in Fe‐deficient wild type plants as compared to the control, but unchanged in the *erfVII* (Figure 4d). Although the two genotypes displayed comparable root length on Fe‐, the mutant resulted insensitive to mild chronic starvation (Figure S3a,b), compatible with higher internal Fe levels. The mutant grew unaffected on sub‐optimal Fe concentrations (15 μM) that, instead, significantly restrained root elongation in the wild type, and only became sensitive to stronger Fe limitation (≤ 5 μM Fe) (Figure S3d,e). The data indicates that the ERFVII take part to the modulation of iron usage or uptake, depending on the severity of Fe‐deficiency conditions. No significant differences were found between genotypes in the expression of the Fe‐inducible transporter genes *IRT1, OPT3* and *NRAMP4*, at the whole seedling level (Figure 4c and Figure S3c).

To further explore the link between hypoxia signaling pathway and Fe starved plants we investigated the ionome profile on the PCO‐defective mutants *pco1/2* and *pco4/5* (Weits et al. 2014, 2023) at the seedling stage. Under Fe‐sufficient conditions, the mineral profiles of the *pco* mutants were largely comparable to the wild type (Col‐0), with only minor fluctuations in elements such as Mg, Ca (increased content in *pco* mutant respect to the wild type), and Mo (decreased content in *pco* mutants). Fe content decreased consistently in Fe‐starved wild type plants, whereas it was less affected in *pco* mutants. Other microelements, such as Mn, Co, and Zn, were accumulated to similar extent in Fe‐starved wild type and *pco* mutant plants, with respect to Fe control conditions, with minor differences between the mutants. Overall, the results suggest that PCOs affect Fe and Mo content, thereby playing a role in fine‐tuning Fe and redox‐related nutrient homeostasis during Fe deficiency stress (Figure S5).

### 3.4 Low‐Oxygen Responses in Control or Fe‐ Conditions

Since the ERFVIIs were found to be connected with Fe starvation responses, we decided to investigate the interplay between Fe starvation and low oxygen conditions, where the ERFVII proteins are known to be stabilized and active. We asked ourselves whether iron availability may contribute to the full extent of hypoxic response in *Arabidopsis* plants and, on the other hand, whether the ERFVII factors may mediate Fe‐starvation responses during hypoxia.

**Figure 5:**
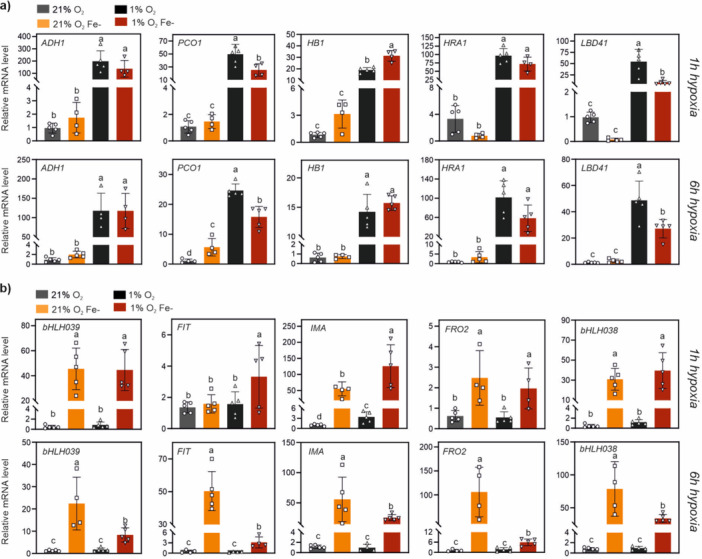
Effect of short or prolonged hypoxia on gene expression in seedlings grown under chronic Fe‐deficiency. Expression of (a) hypoxic and (b) iron‐deficiency marker genes in Col‐0 seedlings grown for 10 days under chronic iron‐deficiency and exposed to short hypoxia (1 h) or prolonged hypoxia (6 h) at 1% O_2_. Histograms show mean ± SD (*n* = 5) of normalized expression values against one Fe‐ctrl aerobic sample. Letters indicate statistically significant difference (*p* < 0.05, two‐way ANOVA, Tukey‐Kramer post hoc test). [Color figure can be viewed at wileyonlinelibrary.com]

Wild type seedlings germinated as described before, on axenic Fe+ or Fe‐ media, were treated with short or prolonged hypoxia (1 or 6 h treatment with 1% O_2_ atmosphere). Aerobic Fe‐ seedlings did not display any up‐regulation of the hypoxic markers (Figure 5a), confirming that PCO catalysis was not affected by moderate Fe‐depletion. Acute hypoxia, instead, triggered hypoxic gene expression, with up to 200‐fold induction, which then progressed in a gene‐specific fashion in the long term of stress. After 1 h, hypoxia marker transcripts were accumulated at the same levels in the two Fe supplementation regimes. This suggests that early marker gene induction, enabled by ERFVII stabilization, depended on quick PCO inactivation exclusively due to lack of sufficient oxygen for catalysis. After 6 h hypoxia, the effect of Fe‐deficiency on hypoxic gene expression remained marginal: Fe‐starved seedlings showed slightly less sustained expression of some markers (*HUP7, PCO1, LBD41*), while the other genes (*Hb1, PDC1, ADH*) were unaffected (Figure 5a). The data thus suggest that plant ability to respond to acute hypoxia stresses cannot be modulated by partial Fe removal from the environment.

We then looked into the regulation of five Fe‐deficiency inducible genes during hypoxia (Figure 5b). In control plants, hypoxia did not promote the induction of any of the starvation markers tested, suggesting that ERFVII stabilization is not sufficient to promote Fe‐deficiency responses. Furthermore, in Fe‐starved seedlings (which as expected showed up‐regulation of the markers, in normoxia), transcripts dropped when plants were shifted to hypoxia for 6 h. Since the hypoxia‐inducible hormone ethylene has been proposed to participate to the induction of Fe‐deficiency responses (Li and Lan 2017; Lucena et al. 2015), we evaluated the effect of hypoxia on the modulation of ethylene biosynthetic gene expression. Fe depletion stimulated the expression of ACC synthase genes (*ACS*), as expected from previous reports (García et al. 2010; Ye et al. 2015); in particular, in full seedlings we observed higher expression under chronic Fe‐depletion conditions of *ACS2* and *ACS9*, but not *ACS7* (Figure S6). ACC oxidase genes (*ACO1* and *ACO2*) were instead unaffected by Fe availability, but *ACO1* showed strong response to 6 h hypoxia. As observed in the case of Fe‐deficiency markers, *ACS2* response to Fe‐deficiency was restrained by hypoxia (Figure S6). This suggests that acute hypoxia might restrict Fe‐deficiency induced transcription by dowregulating ethylene production through *ACS2* repression (Ye et al. 2015).

**Figure 6:**
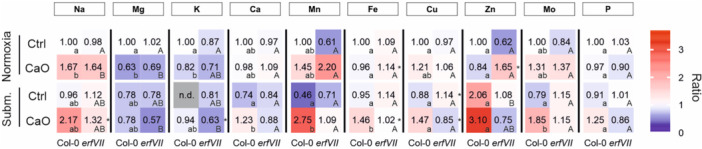
Heatmap of relative ion concentration in Col‐0 and *erfVII* plants grown in control or alkaline and treated with normoxia or submergence conditions. Leaf content of inorganic elements in three week‐old plants grown on control (pH 5.5) or alkaline soil (CaO, pH 7.8) (*n* = 3). Ion abundance was expressed as the ratio to control Col‐0 plants in normoxia and displayed as a heatmap. Ion concentration data are reported in Table S3. Significant differences between the mean ion concentration of Col‐0 and *erfVII* (within conditions) are indicated by an asterisk (*p* < 0.05 after *t*‐test). Different letters indicate significant differences among conditions within genotypes, after two‐way ANOVA followed by Tukey's test (*p* < 0.05). Lower and upper case letters were used for Col‐0 and *erfVII*, respectively. [Color figure can be viewed at wileyonlinelibrary.com]

To reproduce a closer experimental setting to natural flooding combined with nutrient stress, we next investigated the response of soil grown *Arabidopsis* plants to 12 h submergence in presence of normal or alkaline substrate. We used ICP‐MS to evaluate the impact of the interaction between iron starvation and hypoxia on nutrient acquisition. When wild type or *erfVII* plants were grown in normal soil, submergence had a negligible effect on leaf ion content (Figure 6 and Table S3). Instead, a combination of submergence and alkaline conditions enhanced nutrient content (Ca, Mn, Mo and Fe itself) in wild type leaves, but not in the *erfVII*, as compared with control plants. This data suggest that nutrient uptake during the first phases of submergence can be favored by the ERFVII at high soil pH.

We expanded the ICP‐MS analysis to *pco4/5* plants (Figure S7a). We limited the analysis to this mutant, in consideration of the similar behavior displayed by the two *pco* mutants at the seedling stage (Figure S5) and of the functional overlap between constitutive and hypoxia‐inducible *PCO* genes observed in *Arabidopsis* (Weits et al. 2023). As shown by a hierarchical clustering analysis (Figure S7b), under normoxia, the alkaline pH treatment (CaO) separated all genotypes, indicating that soil pH acted as the dominant driver of ionomic variation in well‐aerated plant leaves. Nonetheless, CaO and control ionomic profiles in *pco4/5* and *erfVII* mutants displayed higher divergence than in the wild type, mainly due to variation in Fe, Mg, P, K and Ca, for both *erfVII* (Figure 6) and *pco4/5* (Figure S7a) mutant lines. In contrast, under submergence the responses differed substantially. The *erfVII* mutant clustered away from both the wild type and the *pco4/5* mutant (Figure S7c), whereas the ionomic profiles of CaO and control samples showed comparable responses to Fe‐starvation in wild type and *pco4/5* plants (Figure S7a). These findings reinforce the conclusion that ERFVII stabilization during submergence (expected to occur both in Col‐0 and *pco4/5*, but not in *erfVII*) is important to maintain proper ionomic adjustments in response to Fe‐starvation. At the same time, disruption of the PCO pathway impacted on ion homeostasis in aerated conditions as well, but the similarity of mutant profiles under Fe‐starvation suggest that the dysregulation of ERFVII stability was not the exclusive causative mechanism.

**Figure 7:**
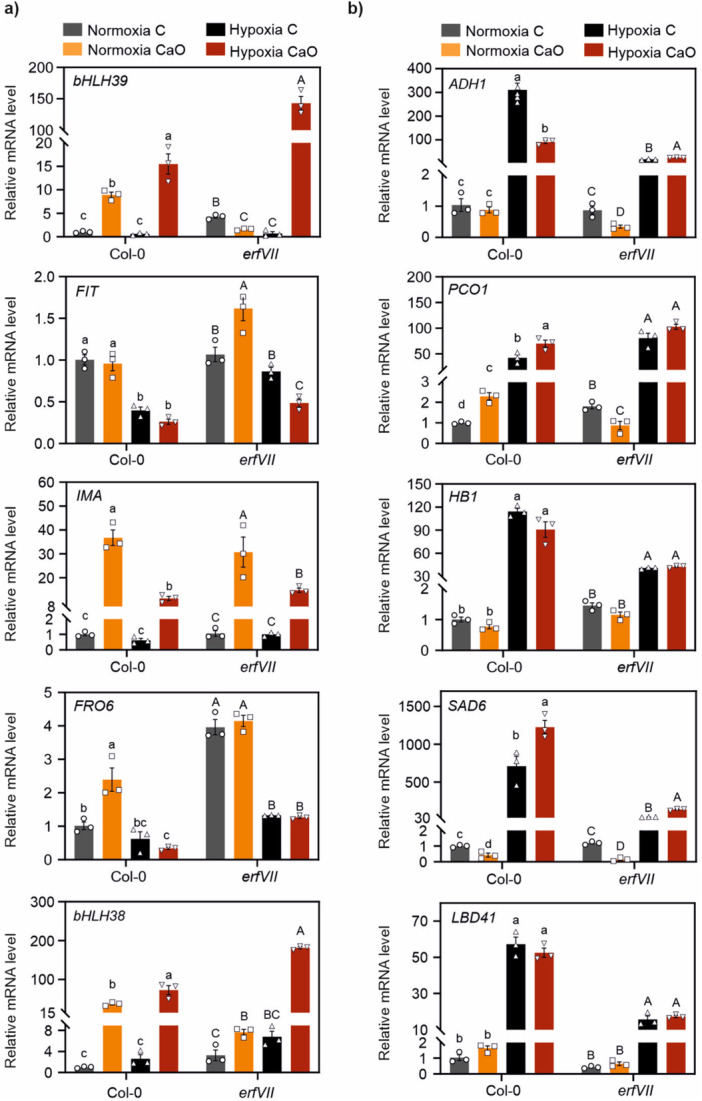
Molecular responses in *erfVII* mutants grown in alkaline soil. Expression of (a) Fe‐deficiency and (b) hypoxic genes in leaves of Col‐0 and *erfVII* plants grown for three weeks in control (pH 5.5) or alkaline soil (CaO, pH 7.8) (*n* = 3). Letters indicate statistically significant differences within the same genotype (*p* < 0.05, two‐way ANOVA, Tukey–Kramer post‐hoc test). [Color figure can be viewed at wileyonlinelibrary.com]

We further explored the role of the ERFVIIs by evaluating changes in marker gene expression during alkaline submergence. CaO supplementation resulted in a moderate alkaline condition (pH 7.8) that, under normoxia, did not alter the total Fe content of leaves in 21‐days old plants (Figure 6), which are prioritized tissues in terms of iron transport (Vigani et al. 2017). Nonetheless, a transcriptional analysis revealed that Fe‐responsive markers except for *FIT* were induced in leaves from the same plants (Figure 7a), indicating the activation of Fe‐deficiency‐induced responses. Soil alkalinization was not sufficient to elicit any hypoxia‐like response in normoxic plants, as expected from the previous observations, nor it impacted on hypoxia marker gene upregulation by submergence (Figure 7b). This suggests that submerged plants growing on moderately alkaline substrates are able to mount normal hypoxic responses (as inferred from core hypoxia‐inducible genes).

Submergence *per se* did not affect the expression of Fe‐deficiency markers (Figure 7a), similar to hypoxia in seedlings (Figure 5). However, submergence combined with alkaline soil put *erfVII* mutant plants into a mild Fe starvation state, not experienced by the wild type. Among the Fe marker genes that displayed significant variations in leaves, *bHLH038* and *039* (which under normoxia had lower expression than in the wild type, as already shown in Figure 3) were indeed more expressed in *erfVII* than Col‐0 plants grown on CaO during submergence (Figure 7a). *IMA* expression, instead, was comparable to the wild type. The stimulation of Fe‐deficiency responses under submergence was in line with lower Fe leaf content in the mutant under combined stresses. Moreover, the differential expression of the tested markers suggests that the ERFVII factors are involved in the regulation of a subset of Fe responsive genes, by restraining their induction in submerged leaves of Fe‐starved plants.

## 4 Discussion

Plants have evolved sophisticated mechanisms to sense and respond to environmental fluctuations, leading to an increase in the frequency and distribution of different stress factors worldwide. The co‐occurrence of multiple stresses may negatively impact plants on growth and health through synergistic or antagonistic interactions among different pathways, networks, and mechanisms that are promoted by each of the stress factors (Zandalinas and Mittler 2022). Here, we examined the molecular cross‐talks between Fe‐deficiency and low oxygen response pathways in the model plant *A. thaliana*, which have received little attention so far.

Fe and hypoxia are expected to be interconnected at two major points in plant physiology: (1) the phytohormone ethylene is a mediator of hypoxia and Fe‐deficiency responses (Li and Lan 2017; Lucena et al. 2015), and (2) non heme Fe‐dependent dioxygenases are responsible for oxygen sensing (Perri and Licausi 2024; White et al. 2017, 2018). In our work, we moved from the latter, asking ourselves whether PCOs might serve as multisensors of Fe^2+^ and O_2_ fluctuations. A main objective of this study was therefore to investigate the sensitivity of PCO enzymes to Fe deprivation, in physiological Fe‐deficiency situations. Several studies indicate that Fe economy in plants involves the differential regulation of the various iron‐requiring enzymes (FeREs) at different stages of severity of Fe‐deficiency stresses (Blaby‐Haas and Merchant 2013; Vigani and Murgia 2018). The preferential use of Fe by specific metabolic pathways under Fe‐deficiency, known as “priority of Fe use”, is believed to serve as an acclimation mechanism to preserve Fe for the most important functions (Hantzis et al. 2018) and represents a hallmark of the phase of resistance to the stress (Vigani and Murgia 2018). By evaluating ERFVII stability and the expression of hypoxic marker genes as proxies of PCO activity (Figures 1 and 4), we could conclude that PCO catalysis is only affected when Fe is left in trace amounts in tissues. This suggests that PCOs might be identified as prioritized components during Fe‐deficiencies, meaning that PCOs engagement might occur later in the progression of Fe‐deficiency induced responses.

The differential engagement of FeREs can be explained by their affinity for Fe as compared with the physiological concentration of free redox‐active Fe ions, also known as Labile Iron Pool or LIP. FeREs characterized by K_mFe(II)_ values close to LIP concentration at a specific subcellular location will be strongly affected by Fe fluctuations, becoming partially or completely inhibited as Fe availability falls below the physiological LIP. In contrast, FeRE with lower K_mFe(II)_ than the physiological LIP may remain active even under Fe‐deficiency (Vigani, Morandini, et al. 2013). The former behavior is expected to be associated with Fe sensing functions. Based on *in silico* predictions, it has been proposed that the plant 2‐OG dependent dioxygenase (2‐ODD) superfamily of O_2_‐dependent FeREs might harbor enzymes with iron sensing potential (Kundu 2015; Vigani, Morandini, et al. 2013). However, such a role has been ruled out for the human prolyl‐4‐hydroxylases (PHDs), 2‐ODD enzymes that serve as oxygen sensors in metazoans. In fact, it has been ascertained that PHD2 can form stable complexes with Fe(II) (Flashman et al. 2010; Hirsilä et al. 2003).

No experimental data of K_mFe(II)_ are available, instead, for thiol dioxygenases. It has been shown that excess of divalent Zn ions can displace Fe(II) from the catalytic site of PCOs and, thereby, lead to the activation of Fe‐deficiency‐like responses in poplar and *Arabidopsis* (Carbonare et al. 2019a). Purified PCO and cysteine dioxygenase enzyme (CDO, a closely related dioxygenase present in metazoans) have been suggested to be unable to bind Fe(II) tightly, based on their binding of substechiometric amounts of the cofactor (Imsand et al. 2012; White et al. 2018). However, exogenous Fe(II) supplementation to the five different purified PCO isoforms of *Arabidopsis* enhanced PCO2 specific activity, but had no effect on the other isoforms. In our *in vivo* Fe‐removal experiments, Fe withdrawal from the media had no effect on reporter stability over 18 h, unless a chelator was added. In parallel, we showed that PCO1 had an observed half‐life above 12 h in *Arabidopsis* seedling tissues (Figure 1b–d). We thus speculate that enough Fe(II) can be coordinated in the catalytic center of PCOs even at very low intracellular Fe concentrations, both in preformed enzymes, which are not likely to lose their cofactor, and in newly synthesized ones.

Among the numerous Cys2 proteins encoded by the *Arabidopsis* proteome, four group Ib bHLH factors can be found, namely bHLH038, 039, 100 and 101. Recently, (Kozlic et al. 2022) detected the marginal stabilization of an (Ala2)bHLH038‐GFP protein expressed in yeast. Here, using a heterologous yeast strategy (Puerta et al. 2019) and transient protoplast transformation, we found that bHLH039 is not a susceptible substrate of the Cys N‐degron pathway (Figure 2). Considering our conclusions on PCO prioritization in terms of Fe usage, we speculate that subtracting the Ib bHLHs from the Cys N‐degron pathway is functional to ensure their accumulation under mild Fe‐deficient conditions. Our bHLH039‐Fluc constructs displayed very low stability, according to the luminescent output, although perfectly detectable bHLH039‐mCherry levels have been reported by (Trofimov et al. 2019) in *Arabidopsis* leaves. The data hint at the presence of some uncharacterized degradation signal in the N‐terminal part of the protein, associated with bHLH039 turnover both in yeast and plant leaf protoplasts, in Fe‐replete conditions.

We investigated the implications of PCO inhibition under severe Fe‐deficiency in light of ERFVII regulation by co‐occurring environmental factors, in addition to oxygen availability. We recorded the transcriptional signature of hypoxic responses in wild type *Arabidopsis* plants growing in chronic Fe‐deficiency conditions, indicating that Fe starvation can affect hypoxia signaling depending on its severity. Moderate Fe stress, either caused by Fe depletion in the media or by plant's acquisition defects, did not invoke hypoxic signaling (Figures 3 and 5, and Figures S3 and S4), while severe chronic Fe‐deficiency did (Figure 4). In axenic seedlings, stabilization of ERFVII factors and hypoxic gene induction in chronic Fe‐deficiency conditions has been reported in previous studies (Dalle Carbonare et al. 2019; Zubrycka et al. 2023). Here, notably, we observed the same response both in seedlings cultivated on synthetic agar media and in adult plants growing on soil, suggesting that the stimulation of hypoxic signaling is part of a general strategy under Fe insufficiency. We cannot rule out that altered oxygen consumption might take part to this phenomenon. Mitochondrial electron transport chain (mETC) complexes are highly dependent on Fe availability and therefore it has been demonstrated that severe Fe‐deficiencies strongly affect mitochondrial functionality both at root and leaf level. Despite the impairment of respiration, increased oxygen consumption has been indeed reported in cucumber roots subjected to severe Fe‐deficiency (Vigani et al. 2009). This effect has been attributed to the increase of other O_2_‐consuming processes, such as Fe^3+^‐chelate reductase and some ROS detoxification activities.

The comparison of wild type and *erfVII* pentuple mutant plants (Abbas et al. 2015) shed light on the contribution of ERFVII stabilization to Fe‐deficiency responses. The *erfVII* mutation was characterized by some alterations in micronutrient homeostasis in full seedlings experiencing moderate starvation and, notably, by better Fe content (Figure 4d). The down‐regulation of some of the Fe‐starvation markers observed in mutant seedlings (Figure 4c) and rosette tissues (Figure 3a), particularly *bHLH038* or *039*, suggests that the ERFVII may contribute to the response to chronic Fe deprivation by positive regulation of a subset of starvation‐responsive genes. Moreover, combination of submergence with alkaline soil highlighted a positive role for the ERFVII factors in the maintenance of adequate nutrient levels in leaves, particularly micronutrients (Figure 6). Accordingly it has been demonstrated that under flooding events, several ions (e.g. Na, Mn, and Fe) accumulate (Setter et al. 2010), while plants can restrict the translocation of toxic ions to the biomass in a genotype‐dependent manner (Kashem and Singh 2001). The relevance of this process to submergence tolerance is supported by the alteration of iron homeostasis in the roots of tolerant *Arabidopsis* ecotypes (Van Veen et al. 2016). In waterlogged soils, pH tends toward neutrality, with increases in acidic soils and decreases in alkaline ones (Husson 2013; Parent et al. 2008) Therefore, the effect of submergence on alkaline soils undergoes a chemical shift toward lower redox potential and pH, setting the stage for affected solubility of some minerals and in turn affecting ionome profile in plants. Our experiments expand the role of ERFVIIs to nutrient management under submergence and dampening of Fe‐starvation responses in the leaves of submerged plants growing on moderately iron‐deficient soil (Figure 7). A role in the negative regulation of the Fe uptake genes has been proposed before for the ERFVII factor RAP2.3 (Liu et al. 2017). Future investigations will clarify the ERFVII target specificity among Fe‐starvation responsive genes and shed light on the molecular mechanisms underlying the alterations in Fe homeostasis (absorption, transport, or usage) observed in the *erfVII* mutant.

A well‐known player in submergence responses is the phytohormone ethylene, which in submerged tissues accumulate rapidly due to a combination of enhanced biosynthesis and restrained outward diffusion (Sasidharan et al. 2018; Sasidharan and Voesenek 2015; Voesenek et al. 1993). On the other hand, transcriptome analyses and genetic evidence indicate that ethylene is also involved in the promotion of Fe uptake in plants through the regulation of FIT (García et al. 2014; Lingam et al. 2011). Ethylene is thus considered to be a positive regulator of Fe‐deficiency responses, during which its synthesis increases (Ye et al. 2015), although full understanding of the underlying mechanisms is yet to be gained (Li and Lan 2017; Lucena et al. 2015). Submergence induced ethylene build‐up has been shown to contribute to the ERFVII‐mediated induction of hypoxic gene expression indirectly, through phytoglobin‐mediated NO scavenging and consequent ERFVII stabilization (Hartman et al. 2019). This mechanism explains the observation that ethylene pretreatment can prime ERFVII‐mediated responses to subsequent hypoxia, in which ethylene entrapment is not expected to take place. We observed a down‐regulation of the transcriptional Fe‐ markers by prolonged hypoxia (Figure 5), which was also present in submerged leaves of Fe‐starved plants (Figure 7). The effect might be due to a global strategy of selective transcriptional prioritization under hypoxia. However, this finding is compatible with a specific function of ERFVII family factors as negative regulators of genes involved in Fe uptake under Fe‐deficiency conditions (Liu et al. 2017). We observed the concurrent down‐regulation of *ACS2* (Figure S6), a required upstream factor in Fe‐ marker gene expression under prolonged Fe‐starvation (Ye et al. 2015). Although downregulation of ethylene biosynthesis is unexpected under hypoxia, this evidence suggests that altered ethylene levels might contribute to shape plant nutritional responses in the presence of combined low oxygen and low Fe conditions. Ethylene and ERFVII signaling may act through parallel mechanisms as well as through cross‐talks. The latter may including the aforementioned NO modulation, or the action of MPK3/6 kinases, which constitute a common node in Fe‐deficiency and low oxygen signaling (Ye et al. 2015; Zhou et al. 2022). Remarkably, use of the *pco4/5* mutant (Weits et al. 2023) highlighted that simple ERFVII stabilization in normoxic conditions was not sufficient to promote the same nutritional adjustments to Fe starvation that could be associated with ERFVII activity under submergence (Figure S7). This reinforces the hypothesis that, beyond the impairment of the Cys N‐degron pathway, other regulatory mechanisms specifically associated with submergence or hypoxia are required to enable ERFVII activity in Fe‐deprivation responses. Future studies will help disentangle the contribution of ethylene‐ and ERFVII‐mediated signaling to nutrient acquisition under combined nutritional and submergence stresses.

It is accepted that flooding tolerance should include traits associated with the alleviation of nutrient deficiencies and phytotoxicity issues that can both arise after prolonged submergence or soil waterlogging (Yuan et al. 2023). Waterlogged soils undergo complex modifications of pH and microbial activities, as a consequence of redox changes, that, depending on the specific edaphic properties, can often lead to the increase of nutrient availability above the toxicity threshold (typically, Zn(II), Mn, Fe(II), or S) (Shabala 2011; Tamang and Fukao 2015). Fe toxicity, for instance, is recognized as a relevant problem in several rice cropping areas of the world (Mahender et al. 2019; Shabala 2011). On the other hand, submergence is expected to impair nutrient acquisition as a consequence of limited transpiration and inhibition of active membrane transport. Reduced mobilization of nutrients to the shoots has been indeed frequently noticed in waterlogged cereals (Colmer and Greenway 2011; Singh and Setter 2017; Steffens et al. 2005). Our finding that plant ERFVII factors participate to nutrient management in plants growing on alkaline substrate might be of potential interest for the improvement of crop responses to waterlogging/submergence events in calcareous and Fe‐deficient soils, which are believed to account to 30% of soils globally (Mahender et al. 2019).

In conclusion, this study provided novel insights into the interactions between low Fe‐ and low O_2_‐response pathways in plants and their implications for plant stress responses. Understanding the intricate regulatory networks that govern Fe homeostasis and O_2_ sensing is crucial for enhancing plant growth, improving stress tolerance, and developing sustainable agricultural practices to withstand the current environmental challenges. Further investigations will be needed to gain a more detailed view of ERFVIIs’ role in fine‐tuning gene expression during chronic Fe‐deficiencies, their impact on acclimation responses to Fe starvation, and their interplay with ethylene, during combined Fe and submergence stresses.

## Conflicts of Interest

The authors declare no conflicts of interest.

## Supporting information


**Supporting Information Figure S1:** Supporting blot images. (a) Full blots from Figure 1e. Lanes 1‐2, 7‐8 and 15‐16 are displayed in the main text. (b) Full blots from Figure 1g. Two biological replicates were performed. **Supporting Information Figure S2:** Effects of prolonged Fe‐depletion on RAP2.12. **Supporting Information Figure S3:** Phenotypic and molecular responses in *erfVII* mutants under moderate chronic iron‐deficiency. **Supporting Information Figure S4:** Expression of Fe‐starvation and hypoxia markers in Fe acquisition mutants. Seedlings were grown for 10 days on agar plates with Fe‐sufficient conditions (50 μM Fe). **Supporting Information Figure S5:** Ion profiles of *pco* mutant seedlings under Fe‐strarvation. Heatmap of relative ion concentrations in *pco1/2* and *pco4/5* mutant seedlings grown for 10 days on control or Fe‐ plates, normalized on the average ion concentration in control Col‐0 plants. **Supporting Information Figure S6:** Expression of ethylene biosynthetic genes. **Supporting Information Figure S7:** Ionomic profiles of Cys N‐degron pathway mutants. **Supporting Information Table S1:** List of qPCR primers used in this study. **Supporting Information Table S2:** Ion quantification in Col‐0, *erfVII* and *pco* double mutant seedlings by ICP‐MS. Absolute concentration of mineral ions (μg g^−1^ dry weight) from *n* = 3 replicates. Raw data supporting Figure 4 in the main text and Supporting Information Figure S5. **Supporting Information Table S3:** Ion quantification in Col‐0, *erfVII* and *pco4/5* rosette leaves by ICP‐MS. Absolute concentration of mineral ions (μg g^‐1^ dry weight) from *n* = 3 replicates. Raw data supporting Figure 6 in the main text and Supporting Information Figure S7. K in submergence control samples was present above the detection limit of the instrument and could not be determined.

## Data Availability

The data that support the findings of this study are available from the corresponding author upon reasonable request.
