# Supplementary material for: Hypoxic and Fe‐Responses are Regulated by the ERFVII Factors and the PCO Branch of the N‐Degron Pathway According to Iron Availability
Source: Plant Cell Environ. 2026 Mar 8;49(6):3363–80. doi: 10.1111/pce.70466 (PMC13136554; doi:10.1111/pce.70466)
Supplement: Supplementary file 1 — Supporting Information Figure S1: Supporting blot images. (a) Full blots from Figure 1e. Lanes 1‐2, 7‐8 and 15‐16 are displayed in the main text. (b) Full blots from Figure 1g. Two biological replicates were performed. Supporting Information Figure S2: Effects of prolonged Fe‐depletion on RAP2.12. Supporting Information Figure S3: Phenotypic and molecular responses in erfVII mutants under moderate chronic iron‐deficiency. Supporting Information Figure S4: Expression of Fe‐starvation and hypoxia markers in Fe acquisition mutants. Seedlings were grown for 10 days on agar plates with Fe‐sufficient conditions (50 μM Fe). Supporting Information Figure S5: Ion profiles of pco mutant seedlings under Fe‐strarvation. Heatmap of relative ion concentrations in pco1/2 and pco4/5 mutant seedlings grown for 10 days on control or Fe‐ plates, normalized on the average ion concentration in control Col‐0 plants. Supporting Information Figure S6: Expression of ethylene biosynthetic genes. Supporting Information Figure S7: Ionomic profiles of Cys N‐degron pathway mutants. Supporting Information Table S1: List of qPCR primers used in this study. Supporting Information Table S2: Ion quantification in Col‐0, erfVII and pco double mutant seedlings by ICP‐MS. Absolute concentration of mineral ions (μg g−1 dry weight) from n = 3 replicates. Raw data supporting Figure 4 in the main text and Supporting Information Figure S5. Supporting Information Table S3: Ion quantification in Col‐0, erfVII and pco4/5 rosette leaves by ICP‐MS. Absolute concentration of mineral ions (μg g‐1 dry weight) from n = 3 replicates. Raw data supporting Figure 6 in the main text and Supporting Information Figure S7. K in submergence control samples was present above the detection limit of the instrument and could not be determined. [file PCE-49-3363-s001.docx]

**Supporting Information to:**

**“Hypoxic and Fe-responses are regulated by the ERFVII factors and the PCO branch of the N-degron pathway according to iron availability”**

Yuri Telara, Moez Maghrebi, Mikel Lavilla, Noemi La Monaca, Giulia Ambrogini, Alessio Sbrana, Sara Delucchi, Pierdomenico Perata, Gianpiero Vigani, Beatrice Giuntoli

**SI content:**

SI Figures S1-S7

SI Tables S1-S3

**Supporting Information Figures**

**
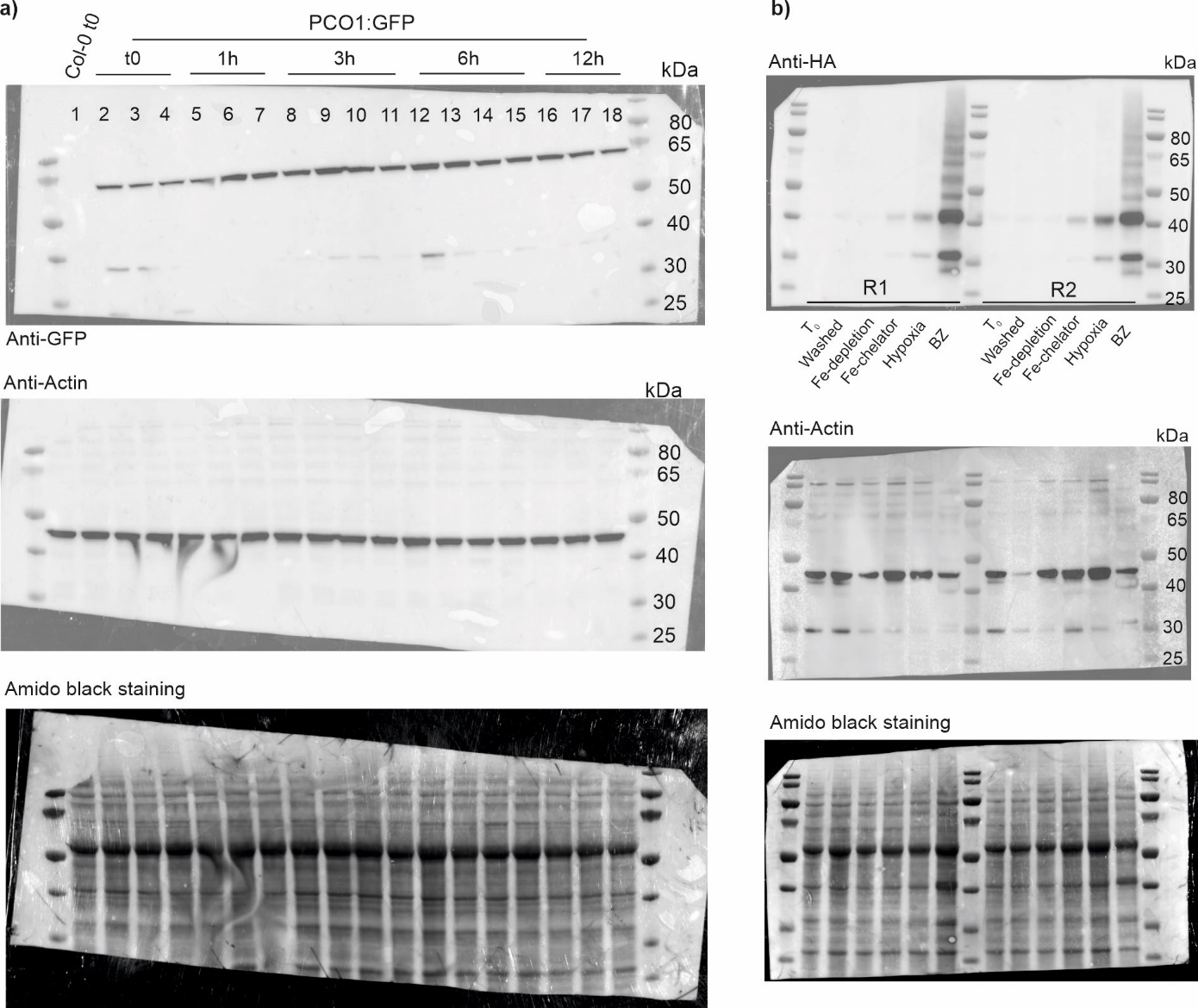
**

**Supporting Information Figure S1. Supporting blot images. (a)** Full blots from Figure 1e. Lanes 1-2, 7-8 and 15-16 are displayed in the main text. **(b)** Full blots from Figure 1g. Two biological replicates were performed.

**
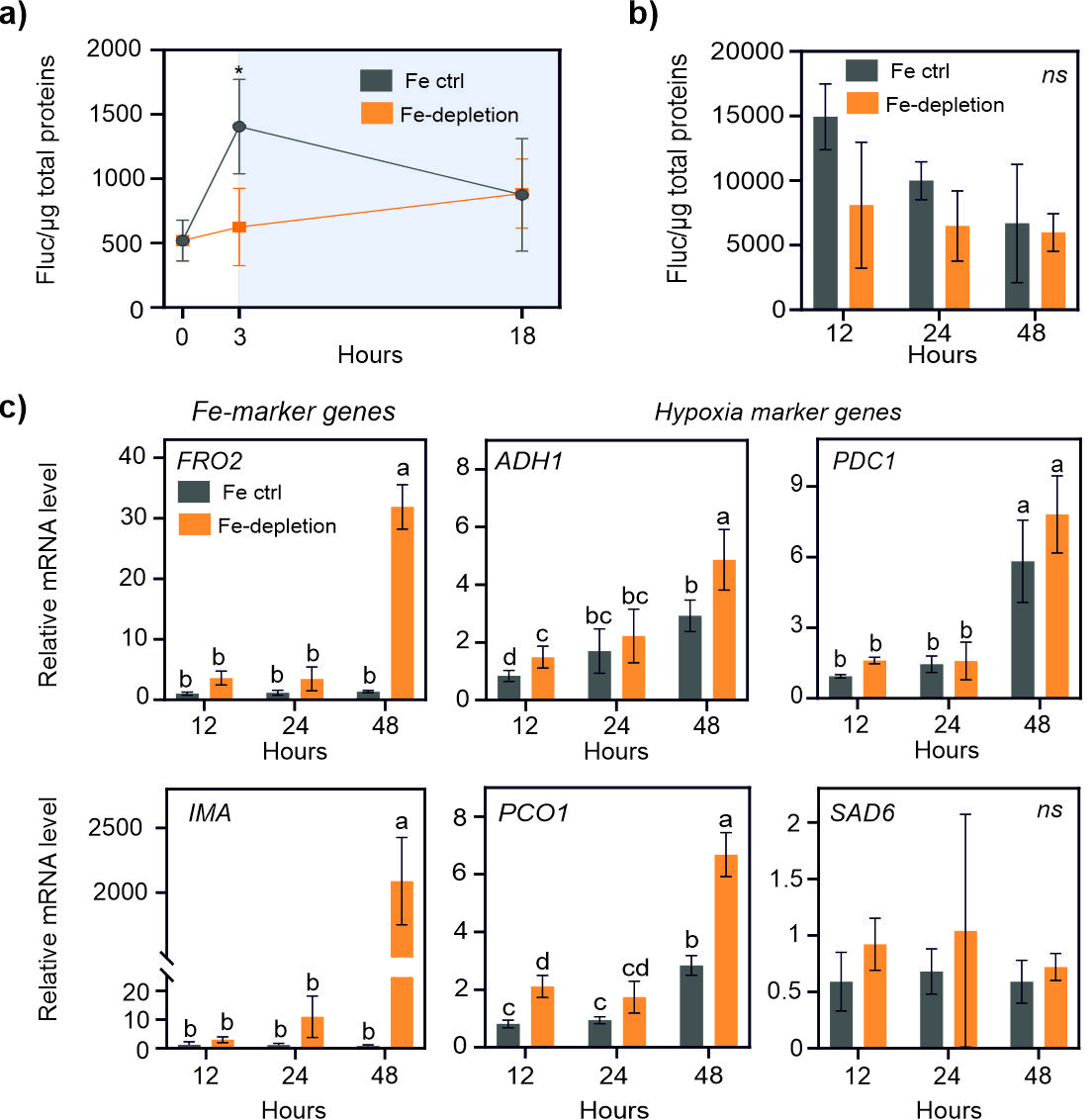
**

**Supporting Information Figure S2. Effects of prolonged Fe-depletion on RAP2.12.** **(a)** RAP2.12 stability in 7 day-old *28RAP2.12Fluc* seedlings after apoplast washing, followed by shifting to fresh Fe-deficiency medium and maintenance under continuous darkness for 3 or 18 h. Data are mean ± SD (n=5). **(b)** 28RAPFluc stability after shifting of seedling to fresh iron-deficiency medium under neutral photoperiod. t_0_ corresponded to the end of day (8 PM). All data are mean ± SD (n=5). **(c)** Expression (mean ± SD, n=5) of marker genes in the Col-0 ecotype, treated as indicated in (b). Asterisks mark statistically significant differences between control and treated samples after Student’s t-test at each time point (P<0.05).

**
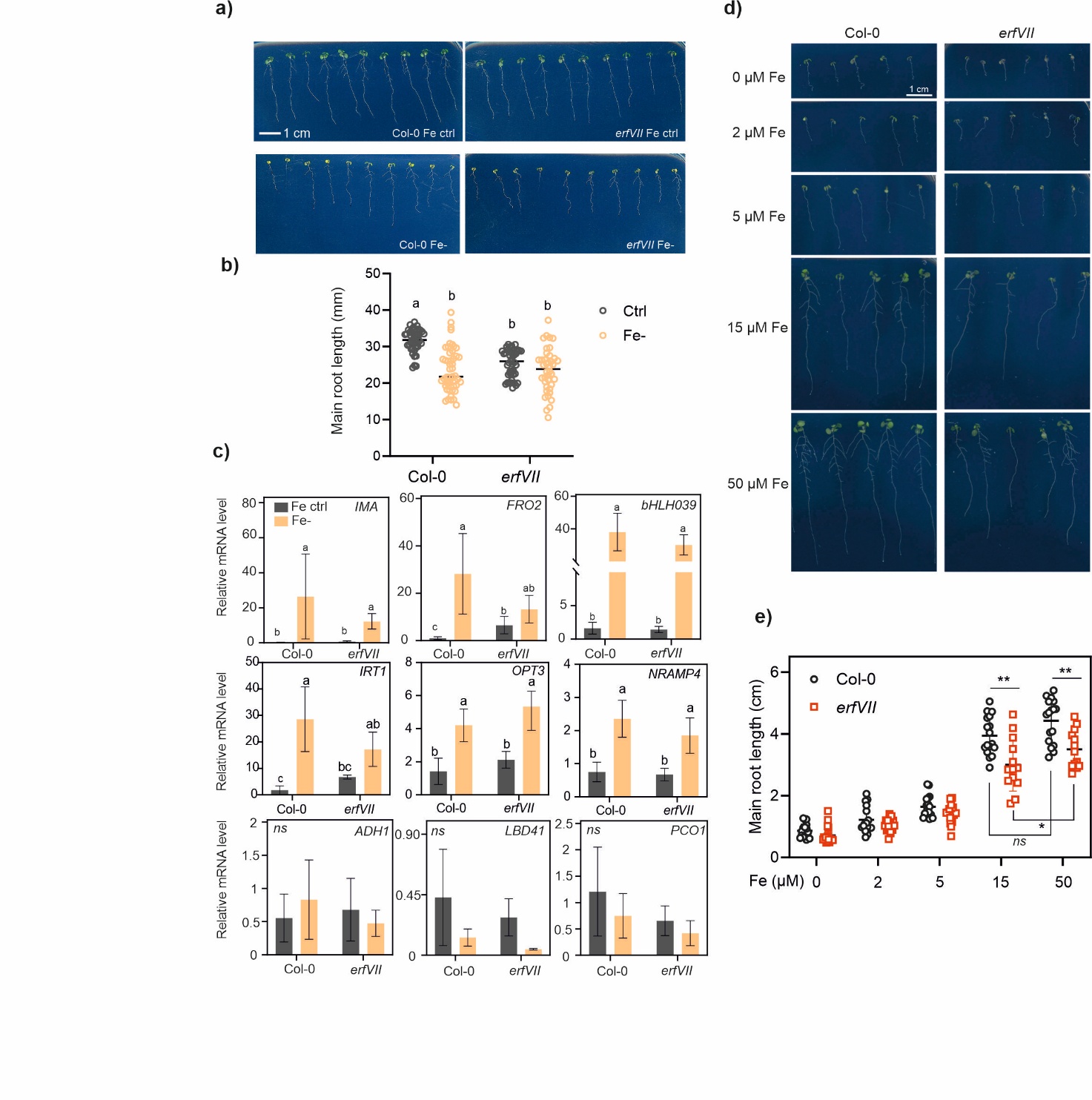
**

**Supporting Information Figure S3. Phenotypic and molecular responses in *erfVII* mutants under moderate chronic iron-deficiency.** **(a)** Representative pictures of wild-type and *erfVII* seedlings grown for 10 days on control or Fe- plates, in presence of 0.9% agar and 1% sucrose (w/v). Five replicate plates were observed for each experimental thesis. **(b)** Quantification of primary root length in the plantlets depicted in (a). Data are mean ± SD (n=57). **(c)** Expression of iron starvation, iron transport and hypoxic markers in the same experiment. Expression values (mean ± SD, n=5) are presented as normalized to a wild-type control sample. Distinct letters indicate statistically significant differences after two-way ANOVA and Tukey-Kramer post-hoc test (P< 0.05). **(d)** Representative phenotypes of 10 day-old seedlings grown in plates on different Fe concentrations, in presence of 0.9% agar and 1% sucrose (w/v). After agar washing with EDTA, Fe was re-supplemented at the specified concentration. **(e)** Primary root length quantification from the previous experiment. Data points (n=13-17) and mean values are displayed. Asterisks above indicate significant differences after t-test within stress levels, comparisons below refer to differences after t-test within genotypes (P<0.05); all not shown comparisons were not significant.


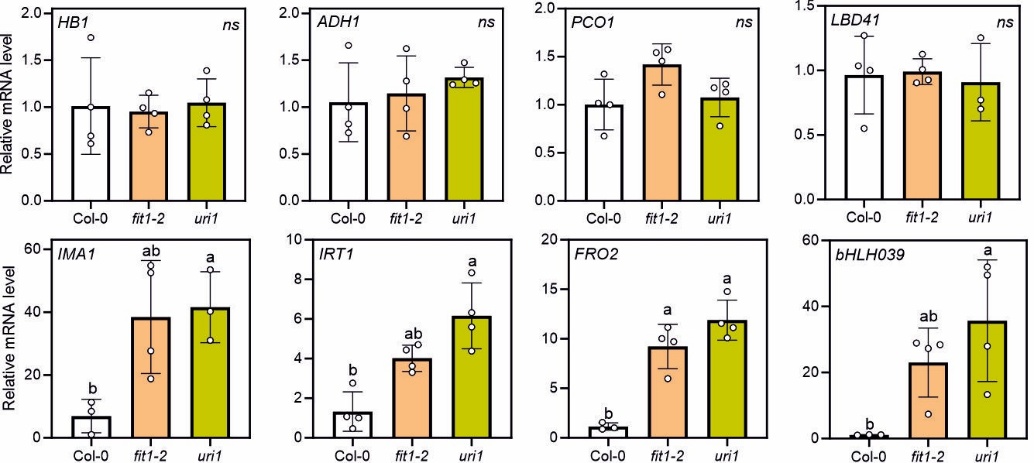


**Supporting Information Figure S4. Expression of Fe-starvation and hypoxia markers in Fe acquisition mutants.** Seedlings were grown for 10 days on agar plates with Fe-sufficient conditions (50 μM Fe). Expression values (mean ± SD, n=3-4) are presented as normalized to a wild-type control sample. Distinct letters indicate statistically significant differences after one-way ANOVA and Tukey post-hoc test (P< 0.05; *ns*, not significant).

**
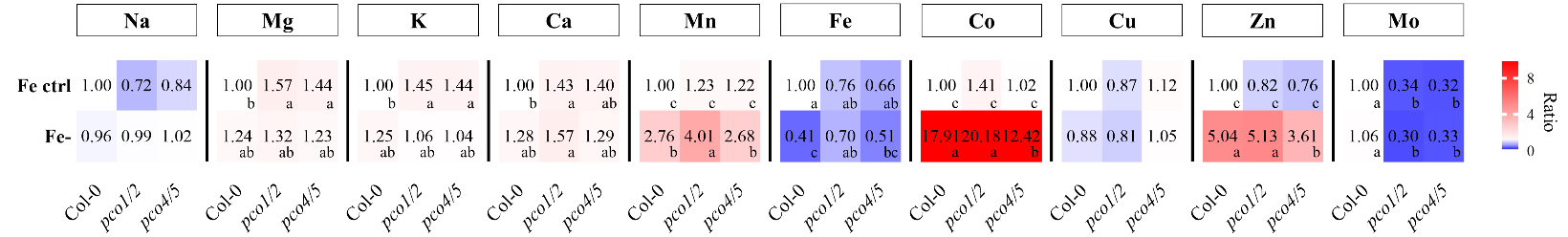
**

**Supporting Information Figure S5. Ion profiles of *pco* mutant seedlings under Fe-strarvation.** Heatmap of relative ion concentrations in *pco1/2* and *pco4/5* mutant seedlings grown for 10 days on control or Fe- plates, normalized on the average ion concentration in control Col-0 plants. Different letters indicate significant differences between conditions and lines (two-way ANOVA, Tukey’s test, P<0.05, n=3). Raw data are provided in **Supporting Information Table S2**.

**
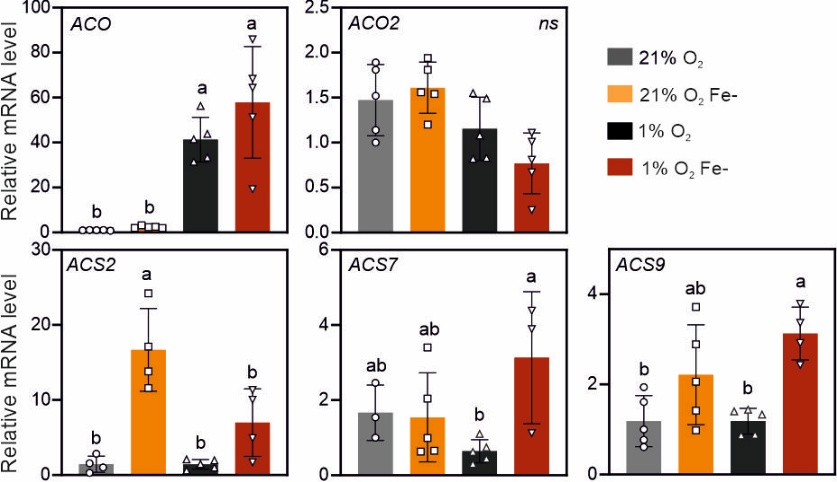
**

**Supporting Information Figure S6. Expression of ethylene biosynthetic genes.** Col-0 seedlings grown for 10 days under chronic iron-deficiency and exposed to short hypoxia (1 h) or prolonged hypoxia (6 h) at 1% O_2_. Histograms show mean ± SD (n=5) of normalized expression values against one Fe-ctrl aerobic sample. Letters indicate statistically significant difference (P< 0.05, two-way ANOVA, Tukey-Kramer post hoc test).

**
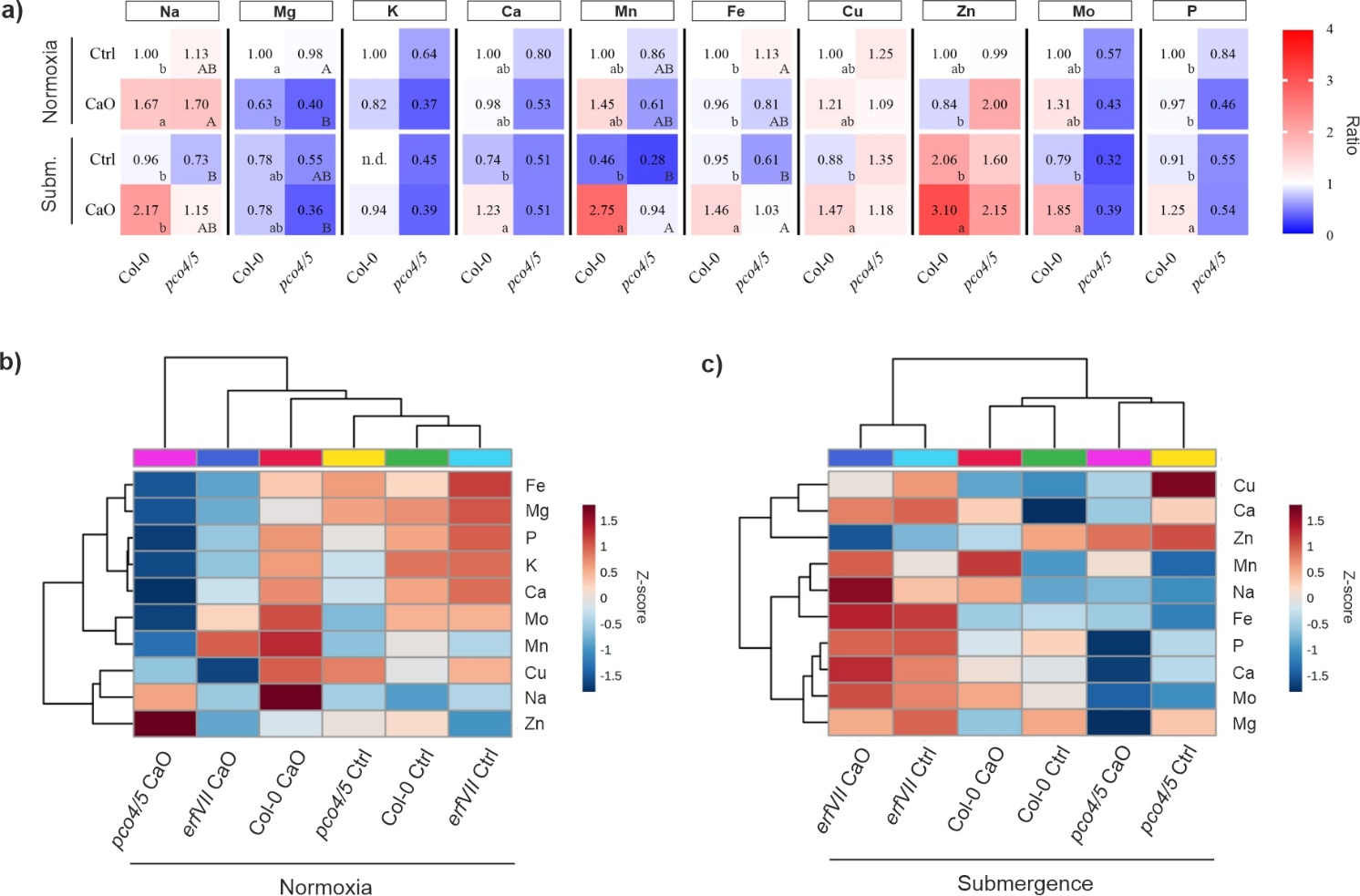
**

**Supporting Information Figure S7. Ionomic profiles of Cys N-degron pathway mutants. (a)** Leaf content of inorganic elements in three week-old *pco4/5* plants, compared to Col-0, grown on control (Ctrl) or alkaline soil (CaO). Ion abundance was expressed as the ratio to control Col-0 plants in normoxia and displayed as a heatmap (n=3). Ion concentration data are reported in **Supporting Information Table S3.** Different letters indicate significant differences among conditions within genotypes after one-way ANOVA followed by Tukey’s test (P<0.05). **(b)** Hierarchical clustering of ion abundance data in normoxic or **(c)** submergence conditions.

**Supporting Information Tables**

**Supporting Information Table S1. List of qPCR primers used in this study.**

| **Gene name** | **AGI code** | **Forward primer sequence** | **Reverse primer sequence** |
| --- | --- | --- | --- |
| *ACO1* | *AT2G19590* | ACCTCAGATGCAGATTGGGAAAGC | CCATCGTCTTGCTGAGTTCCTCTG |
| *ACO2* | *AT1G62380* | TACGTTCGTCACCTCCCTCAATC | GTCTTTCATGGCCGTCCTGTATTC |
| *ACS2* | *AT1G01480* | CAGAGACTTAGAGGCTGTGTTTGC | TCAGACATTTGCTCTCCCAACGTC |
| *ACS7* | *AT2G26200* | GATGGAGAACCGGAGTGAAA | GGTTCGATGGGTTGGTTATG |
| *ACS9* | *AT3G49700* | ACCGTTTTTGGGTTTGAACA | CCTACGCGAAAACCTGGTAA |
| *ADH1* | *AT1G77120* | TATTCGATGCAAAGCTGCTGTG | CGAACTTCGTGTTTCTGCGGT |
| *BHLH038* | *AT3G56970* | TCAACGGTTTCTGCCACTAG | ACATCCACAAGAACAAACCCA |
| *BHLH039* | *AT3G56980* | TGTTTCTGTTTCGTCGGAGG | TAATTTTCCTGCGACGGTCA |
| *FRO2* | *AT1G01580* | GCTTCCGCCGATTTCTTAAGGC | AACGGAGTTATCCCGCTTCCTC |
| *FIT* | *AT1G61360* | GCGGTATCAATCCTCCTGCT | GATGGAGCAACACCTTCTCCT |
| *FRO6* | *AT5G49730* | GTCTAGTGGCGGGTTTACTG | TCACCA ACATGAAGTGCCAA |
| *HB1* | *AT2G16060* | TTTGAGGTGGCCAAGTATGCA | TGATCATAAGCCTGACCCCAA |
| *HRA1* | *AT3G10040* | ACAACCACCGCAACAGAATCC | TCTCCGCAATTCTCGCCAT |
| *IMA* | *AT1G47400* | GGCCATCAAGAGATTTGACCATGC | TGCCACTCGAGAACTATCTACCAC |
| *IRT1* | *AT4G19690* | ACTTCAACTGCGCCGGAAGAATG | AGCTTTGTTGACGCACGGGTTC |
| *LBD41* | *AT3G02550* | TGAAGCGCAAGCTAACGCA | ATCCCAGGACGAAGGTGATTG |
| *NRAMP4* | *AT5g67330* | TGGCTCTTGCATTTGCTTGGATG | AGCTTAGGGACCAATGCTCCAAC |
| *OPT3* | *AT4G16370* | GGAGGCATGTACCGTAACCTTGTC | CCCTGCAAAGCCGTAGGAGATAAC |
| *PCO1* | *AT5G15120* | ATTGGGTGGTTGATGCTCCAATG | ATGCATGTTCCCGCCATCTTC |
| *SAD6* | *AT1G43800* | TTGGCAACCCGCTTCTTTCTTACC | TTTCCCTCAGCTCACGAACCTG |
| *UBQ10* | *AT4G05320* | GGCCTTGTATAATCCCTGATGAATAAG | AAAGAGATAACAGGAACGGAAACATAGT |

**Supporting Information Table S2. Ion quantification in Col-0, *erfVII* and *pco* double mutant seedlings by ICP-MS.** Absolute concentration of mineral ions (μg g^-1^ dry weight) from n=3 replicates. Raw data supporting Figure 4 in the main text and Supporting Information Figure S5.

|  | **Col-0 Fe ctrl** | ***erfVII* Fe ctrl** | | ***pco1/2* Fe ctrl** | | ***pco4/5* Fe ctrl** | |  |
| --- | --- | --- | --- | --- | --- | --- | --- | --- |
| Na | 3571.45±1746.57 | 3341.4±2156.2 | | 2558.73±390.43 | | 3016.47±163.78 | |  |
| Mg | 1834.33±280.24 | 2291.29±217.01 | | 2888.38±494.45 | | 2634.68±29.39 | |  |
| K | 44497.57±7208.97 | 47032.85±3622.96 | | 64380.74±10273.4 | | 64137.55±1059.71 | |  |
| Ca | 4731.82±833.24 | 5791.48±541.23 | | 6756.96±1115.39 | | 6636.39±160.45 | |  |
| Mn | 133.51±21.06 | 137.27±11.16 | | 163.88±13.08 | | 162.97±5.99 | |  |
| Fe | 229.46±59.31 | 194.59±38.4 | | 174.83±27.43 | | 150.54±10.95 | |  |
| Co | 1.32±0.29 | 1.44±0.36 | | 1.85±0.24 | | 1.34±0.13 | |  |
| Cu | 13.63±0.56 | 5.61±1.58 | | 11.86±1.91 | | 15.31±3.15 | |  |
| Zn | 198.61±39.79 | 154.87±38.7 | | 162.23±13.92 | | 150.01±3.2 | |  |
| Mo | 41.84±1.23 | 11.19±2.32 | | 14.4±1.33 | | 13.58±1.27 | |  |
|  | **Col-0 Fe-** | | ***erfVII* Fe-** | | ***pco1/2* Fe-** | | ***pco4/5* Fe-** | |
| Na | 3440.16±1103.19 | | 6822.91±1656.57 | | 3528.17±921.70 | | 3630.20±634.10 | |
| Mg | 2270.85±361.91 | | 3096.55±149.61 | | 2427.07±274.32 | | 2260.08±393.95 | |
| K | 55482.42±11273.34 | | 57659.21±5181.58 | | 47388.23±6676.84 | | 46256±6630.67 | |
| Ca | 6068.61±852.65 | | 8274.12±970.8 | | 7423.26±758.39 | | 6124.24±1035.41 | |
| Mn | 368.48±18.1 | | 532.52±66.38 | | 535.15±41.3 | | 357.95±25.26 | |
| Fe | 94.89±14.1 | | 164.4±8.83 | | 160.56±44.43 | | 118.11±12.56 | |
| Co | 23.58±5.04 | | 23.07±5.67 | | 26.57±2.16 | | 16.36±1.12 | |
| Cu | 12±5.32 | | 17.1±2.2 | | 10.99±1.95 | | 14.32±1.42 | |
| Zn | 1000.21±38.46 | | 992.63±145.02 | | 1018.83±90.87 | | 716.8±88.82 | |
| Mo | 44.46±3.75 | | 132.34±16.69 | | 12.63±1.54 | | 13.84±1.56 | |

**Supporting Information Table S3. Ion quantification in Col-0, *erfVII* and *pco4/5* rosette leaves by ICP-MS.** Absolute concentration of mineral ions (μg g^-1^ dry weight) from n=3 replicates. Raw data supporting Figure 6 in the main text and Supporting Information Figure S7. K in submergence control samples was present above the detection limit of the instrument and could not be determined.

|  | **Col-0** | | | |
| --- | --- | --- | --- | --- |
| **Ion** | **Normoxia ctrl** | **Normoxia CaO** | **Submergence ctrl** | **Submergence CaO** |
| Na | 1631.17 ± 126.76 | 2716.72 ± 91.85 | 1562.13 ± 403.16 | 3546.48 ± 686.34 |
| Mg | 11182.46 ± 1653.93 | 7066.47 ± 147.86 | 8683.28 ± 1343.22 | 8721.52 ± 1718.04 |
| K | 47933.4 ± 7319.26 | 39483.85 ± 4026.18 | over | 44879.21 ± 9150.63 |
| Ca | 44684.27 ± 7394.68 | 43688.52 ± 1911.98 | 32861.91 ± 4844.67 | 54890.25 ± 10168.33 |
| Mn | 96.85 ± 68.51 | 140.26 ± 15.56 | 44.1 ± 6.68 | 266.08 ± 137.24 |
| Fe | 205.44 ± 12.44 | 196.98 ± 5.29 | 194.99 ± 23.7 | 300.02 ± 53.68 |
| Cu | 9.9 ± 1.01 | 12.01 ± 3.67 | 8.69 ± 0.91 | 14.58 ± 2.57 |
| Zn | 141.12 ± 40.68 | 118.92 ± 8.12 | 290.69 ± 214.11 | 437.45 ± 459.88 |
| Mo | 18.33 ± 4.14 | 24 ± 5.99 | 14.53 ± 2.92 | 33.9 ± 12.55 |
| P | 11.63 ± 1.59 | 11.24 ± 0.62 | 10.54 ± 1.34 | 14.54 ± 3.16 |
|  | ***erfVII*** | | | |
| **Ion** | **Normoxia ctrl** | **Normoxia CaO** | **Submergence ctrl** | **Submergence CaO** |
| Na | 1594.22 ± 373.6 | 2667.76 ± 549.28 | 1825.78 ± 415.66 | 2152.56 ± 82.14 |
| Mg | 11355.15 ± 1435.02 | 7709.15 ± 1233.38 | 8721.03 ± 1519.49 | 6416.53 ± 1356.52 |
| K | 41930.21 ± 3325.43 | 34210.44 ± 7012.78 | 38960.89 ± 2412.41 | 30025.99 ± 1361.63 |
| Ca | 43292.64 ± 4745.24 | 48787.99 ± 8624.49 | 37522.87 ± 1434.49 | 39229.35 ± 9194.66 |
| Mn | 59.45 ± 1.59 | 212.99 ± 112.04 | 68.42 ± 24.91 | 105.82 ± 36.66 |
| Fe | 224.29 ± 35.31 | 234.44 ± 3.24 | 233.99 ± 22.19 | 208.82 ± 18.27 |
| Cu | 9.62 ± 0.52 | 10.46 ± 2.19 | 11.27 ± 0.92 | 8.37 ± 1.68 |
| Zn | 87.8 ± 4.94 | 87.07 ± 5.28 | 153.04 ± 39.79 | 106.42 ± 17.24 |
| Mo | 15.47 ± 1.71 | 25.13 ± 11.22 | 21.01 ± 8.78 | 20.98 ± 2.39 |
| P | 11.99 ± 0.62 | 10.43 ± 2.53 | 11.8 ± 0.53 | 10.04 ± 1.68 |

|  | ***pco4/5*** | | | |
| --- | --- | --- | --- | --- |
| **Ion** | **Normoxia ctrl** | **Normoxia CaO** | **Submergence ctrl** | **Submergence CaO** |
| Na | 1842.64 ± 254.36 | 2770.51 ± 220.31 | 1190.97 ± 423.49 | 1870.72 ± 508.81 |
| Mg | 11001.66 ± 1876.8 | 4470.31 ± 998.33 | 6161.24 ± 878.06 | 4066.82 ± 1405.67 |
| K | 30717.67 ±10618.75 | 17532.92 ± 4754.05 | 21472.55 ± 3919.39 | 18459.67 ± 7096.04 |
| Ca | 35862.86 ±10570.21 | 23794.73 ± 6734.61 | 22913.92 ± 2795.77 | 22701.43 ± 7501.81 |
| Mn | 83.45 ± 50.16 | 59.03 ± 16.72 | 26.91 ± 5.14 | 91.28 ± 4.57 |
| Fe | 232.97 ± 20.01 | 166.81 ± 23.27 | 124.91 ± 16.59 | 210.6 ± 8.99 |
| Cu | 12.38 ± 1.67 | 10.76 ± 1.05 | 13.39 ± 2.99 | 11.64 ± 2.52 |
| Zn | 139.16 ± 28.18 | 282.41 ± 89.1 | 225.99 ± 63.43 | 303.63 ± 56.23 |
| Mo | 10.42 ± 3.65 | 7.79 ± 4.43 | 5.9 ± 1.35 | 7.18 ± 2.89 |
| P | 9.72 ± 2.94 | 5.4 ± 1.71 | 6.4 ± 0.7 | 6.24 ± 2.45 |
